# Entry of Polarized Effector Cells into Quiescence Forces HIV Latency

**DOI:** 10.1128/mBio.00337-19

**Published:** 2019-03-26

**Authors:** Curtis Dobrowolski, Saba Valadkhan, Amy C. Graham, Meenakshi Shukla, Angela Ciuffi, Amalio Telenti, Jonathan Karn

**Affiliations:** a Department of Molecular Biology and Microbiology, Case Western Reserve University School of Medicine, Cleveland, Ohio, USA; b Institute of Microbiology, Lausanne University Hospital and University of Lausanne, Lausanne, Switzerland; c Department of Integrative Structural and Computational Biology, The Scripps Research Institute, La Jolla, California, USA; Columbia University; Gladstone Institutes University of California San Francisco; Pennsylvania State University; UCSD School of Medicine

**Keywords:** cell quiescence, HIV latency, HIV reservoir, P-TEFb, human immunodeficiency virus

## Abstract

The latent HIV reservoir is generated following HIV infection of activated effector CD4 T cells, which then transition to a memory phenotype. Here, we describe an *ex vivo* method, called QUECEL (quiescent effector cell latency), that mimics this process efficiently and allows production of large numbers of latently infected CD4^+^ T cells. Naïve CD4^+^ T cells were polarized into the four major T cell subsets (Th1, Th2, Th17, and Treg) and subsequently infected with a single-round reporter virus which expressed GFP/CD8a. The infected cells were purified and coerced into quiescence using a defined cocktail of cytokines, including tumor growth factor beta, interleukin-10 (IL-10), and IL-8, producing a homogeneous population of latently infected cells. Flow cytometry and transcriptome sequencing (RNA-Seq) demonstrated that the cells maintained the correct polarization phenotypes and had withdrawn from the cell cycle. Key pathways and gene sets enriched during transition from quiescence to reactivation include E2F targets, G_2_M checkpoint, estrogen response late gene expression, and c-myc targets. Reactivation of HIV by latency-reversing agents (LRAs) closely mimics RNA induction profiles seen in cells from well-suppressed HIV patient samples using the envelope detection of *in vitro* transcription sequencing (EDITS) assay. Since homogeneous populations of latently infected cells can be recovered, the QUECEL model has an excellent signal-to-noise ratio and has been extremely consistent and reproducible in numerous experiments performed during the last 4 years. The ease, efficiency, and accuracy of the mimicking of physiological conditions make the QUECEL model a robust and reproducible tool to study the molecular mechanisms underlying HIV latency.

## Abstract (precis)

Current primary cell models for HIV latency correlate poorly with the reactivation behavior of patient cells. We have developed a new model, called QUECEL, which generates a large and homogenous population of latently infected CD4^+^ memory cells. By purifying HIV-infected cells and inducing cell quiescence with a defined cocktail of cytokines, we have eliminated the largest problems with previous primary cell models of HIV latency: variable infection levels, ill-defined polarization states, and inefficient shutdown of cellular transcription. Latency reversal in the QUECEL model by a wide range of agents correlates strongly with RNA induction in patient samples. This scalable and highly reproducible model of HIV latency will permit detailed analysis of cellular mechanisms controlling HIV latency and reactivation.

## INTRODUCTION

Patients adhering to combination antiretroviral (cART) regimens have minimal viremia, but HIV persists due to a pool of transcriptionally silenced but replication-competent proviruses found in a small population of resting memory CD4^+^ T cells (1 to 100 per 10^6^ cells) accumulating in the peripheral blood (1) and tissues (2). Since silenced proviruses produce only minimal viral RNA and proteins, they are refractory to antiviral drugs and effectively evade immune surveillance.

Recent studies of proviral integration sites have provided strong evidence for the clonal expansion of memory T cells carrying silenced proviruses (3, 4). These data suggest that the reservoir is in a pseudo-steady state with persistent low-level rates of viral reactivation and cell death counterbalanced by homeostatic expansion of latent clones (3–6). These combined processes result in a very stable reservoir with an apparent half-life of 44 months in the presence of cART (1, 7, 8). The persistent reservoir almost invariably rebounds within 2 to 8 weeks when cART is interrupted (9, 10). HIV latency therefore remains the major obstacle to a functional cure for HIV infections. An attractive approach for eradicating the latent reservoir is the “shock-and-kill” strategy. In this strategy, latency-reversing agents (LRAs) (11, 12) are used to make latently infected cells visible to the immune system (shock) and target them for elimination (kill).

It is currently unknown whether HIV targets activated effector CD4 T cells, which then enter quiescence after the removal of the immune stimuli, and their transition to a memory phenotype (13, 14), or whether HIV enters already quiescent cells (15, 16). Analysis of HIV transcription in resting cells demonstrates that quiescence leads to suppression of both the initiation of HIV transcription and its elongation (17). Several key transcription initiation factors, including NF-κB and NFAT, are sequestered in the cytoplasm of quiescent cells (17). The positive transcription elongation factor b (P-TEFb), which is an essential host cofactor for the HIV transactivator protein, Tat, is also absent from quiescent cells due to degradation of the CycT1 subunit (18–21). Additionally, epigenetic silencing events ensure that proviral latency is maintained (17). T-cell receptor activation reverses each of these restrictions and stimulates HIV transcription, replication, and spread. One reason why the switch to productive transcription when resting cells are activated is so efficient is that multiple signaling pathways are activated, and HIV transcription is selectively enhanced by a positive feedback mechanism fueled by the viral *trans*-activator protein Tat and P-TEFb (17).

Although latency can be established in transformed T cell lines, such as Jurkat T cells (22–26), where P-TEFb is constitutively activated (27), this can lead to potentially unrepresentative mechanisms of HIV latency (26, 28). A more physiologically relevant approach is to infect primary cells isolated from healthy donors (29, 30), and a wide variety of primary cell models of HIV latency have been developed (14, 29–33). Unfortunately, results in these models typically do not correlate well between themselves and with RNA induction measurements in patient cells (26).

Bosques and Planelles (30, 34, 35) introduced methods for infection of an effector cell population derived from naive T cells that is then allowed to enter quiescence. Based on their work, we have developed a refined polarized cell model of HIV latency, called the QUECEL (quiescent effector cell latency) model. A key innovation in the QUECEL model is the use of a defined cocktail of cytokines, including tumor growth factor beta (TGF-β), to force cells into quiescence and yield a highly enriched latent HIV-infected cell population. The QUECEL model has proved to be highly consistent and reproducible in numerous experiments performed during the last 4 years, and activation of HIV by LRAs closely mimics RNA induction profiles seen in cells from well-suppressed HIV patient samples.

## RESULTS

### QUECEL model for HIV latency in primary cell helper subsets.

**FIG 1:**
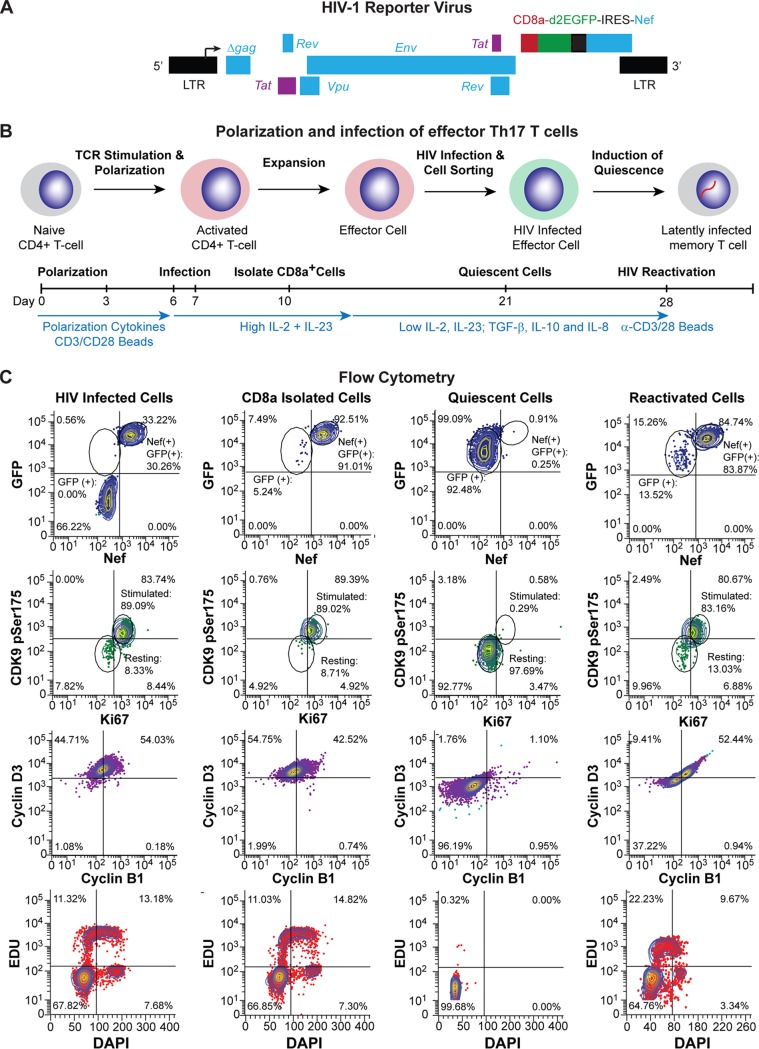
Overview of HIV latency model in effector CD4 T cells. (A) Genome organization of the HIV-1 pNL4-3 delta gag CD8a/GFP-IRES-Nef reporter virus. (B) Method. Naïve T cells were isolated from healthy donors and stimulated through the TCR or by ConA. Activated CD4^+^ T cells were polarized into specific effector CD4^+^ T cell subsets with cytokine cocktails at day 0. Effector cells were infected at day 7 with HIV-GFP/CD8a, sorted by magnetic bead separation, and allowed to enter quiescence, resulting in latently infected cells. Important steps in protocol and relative cytokine levels are indicated on the timeline. (C) Flow cytometry. Expression of HIV Nef, GFP, Ki67, CDK9 pSer175, cyclin D3, and cyclin B1, EDU incorporation, and DNA levels were monitored at each stage of the protocol.

The QUECEL (quiescent effector cell latency) model (Fig. 1) is a refinement of the model of Bosque and Planelles (30, 35). Briefly, naive helper T cells are activated, polarized, and then infected using a single-round vesicular stomatitis virus (VSV)-pseudotyped reporter virus (Fig. 1A) (22, 23, 36). The reporter carries a CD8-enhanced green fluorescent protein (EGFP) fusion protein that permits purification of infected cells with anti-CD8 magnetic beads. Three days after infection, CD8a-expressing cells were isolated and then allowed to expand up to 50-fold for 7 to 10 days.

To induce quiescence, the expansion cytokines were reduced 4-fold to simulate natural cytokine conditions developed *in vivo* in the absence of inflammation (37), and a silencing cocktail containing TGF-β, interleukin-10 (IL-10), and IL-8 was added (Fig. 1B). Expression of the cellular activation markers CDK9 pSer175 (P-TEFb) (38, 39) and Ki67 (14, 26) was high during the expansion phase, while the quiescence conditions resulted in a dramatic decrease of CDK9 pSer175 and Ki67. Cyclin D3 and B1 levels were also reduced drastically upon cellular quiescence, indicative of the cells leaving the cell cycle. This was confirmed by checking EDU incorporation and total DNA content using 4′,6-diamidino-2-phenylindole (DAPI) (Fig. 1C).

Once in quiescence, the level of HIV Nef protein expression is reduced to almost undetectable levels (1%), which is indicative of HIV latency. GFP levels also decline, but because of the high stability of the CD8a-GFP fusion protein (see Fig. S1A and B in the supplemental material), GFP persists in the quiescent cells, albeit at a lower intensity (Fig. 1C). Upon stimulation through the T cell receptor (TCR) (α-CD3/α-CD28 Dynal magnetic beads), the vast majority of cells (>82%) express Nef, and there is a concomitant increase in GFP, Ki67, cyclin B1, cyclin D3, and CDK9 pSer175 levels (Fig. 1C). HIV RNA is also detected in the majority of reactivated cells by RNA fluorescent in situ hybridization (FISH) (Fig. S1C). A useful variation of the QUECEL protocol includes the use of Thy1.2 for purification of the infected cell population and replacement of the CD8a-GFP reporter with a GFP or red fluorescent protein (RFP) reporter (Fig. S1D). The experimental basis for each of the steps in this protocol are described in detail below. For most of the experiments that follow, we have concentrated on the Th17-polarized cells, since there is very limited variation between the different effector subtypes and the Th17 cells have slightly higher viability.

10.1128/mBio.00337-19.1 FIG S1 **CD8a/GFP persists in quiescent QUECEL cells.** (A) Flow cytometry analysis of d2EGFP levels in Th17 cells infected with HIV expressing either the membrane-bound CD8a/GFP fusion protein or cytoplasmic d2EGFP. Cells were analyzed before and after treatment with 10 μg/ml cycloheximide (24 h). (B) Half-life of CD8a/GFP fusion protein and Nef during the establishment of quiescence. Solid lines, positive cells (%); dashed lines, mean fluorsescent intensity. (C) Immunofluorescence and RNA FISH analysis of Th17 cells reactivated through the TCR. Yellow, RNA detected by Stellaris RNA FISH (Biosearch Technologies) using probes spanning the U3 and U5 regions of the provirus. Green, GFP. Blue, DAPI. (D) Flow cytometry analysis of d2EGFP in Th17 cells latently infected with an HIV lentiviral vector expressing a Thy1.2-P2A-d2EGFP reporter gene (top), which permits decay of the cytoplasmic d2EGFP signal during quiescence. (Bottom) d2EGFP induction after stimulation by the TCR, 500 nM SAHA, and 100 ng/ml TNF-α. Download FIG S1, TIF file, 3.3 MB. Copyright © 2019 Dobrowolski et al. 2019 Dobrowolski et al. This content is distributed under the terms of the Creative Commons Attribution 4.0 International license.

### Efficient *ex vivo* polarization of Th1, Th2, Th17, and Treg effector CD4 T cell subsets.

A series of *in vitro* polarization conditions were developed to generate the four most abundant effector CD4 T cell subsets: Th1, Th2, Th17, and Treg (Fig. 1B, Table 1, and the supplemental material). For the Th1 and Th2 subsets, naive CD4 T cells were stimulated through the TCR, and unwanted cytokines were neutralized with monoclonal antibodies to IL-4, IL-12, or gamma interferon (IFN-γ) (40). Th17 cells were polarized in the presence of TGF-β, neutralizing antibodies to IL-4, and the cytokines IFN-γ, IL-6, IL-1β, and IL-23 (41, 42). Regulatory CD4 T cells were generated using TGF-β and neutralizing antibodies to IL-4 and IFN-γ (30).

**TABLE 1:** Polarization conditions

CD4 T cell subset	Cytokine	Concn (ng/ml)	Antibody	Antibody concn (µg/ml)
T helper 1	TGF-β	5	α-Human IL-4	500
T helper 2	TGF-β	5	α-Human IFN-γ	500
T helper 17	TGF-β	5	α-Human IL-4	500
	IL-23	50	α-Human IFN-γ	500
	IL-6	10		
T regulatory cells	TGF-β	5	α-Human IL-4	500
			α-Human IFN-γ	500

**FIG 2:**
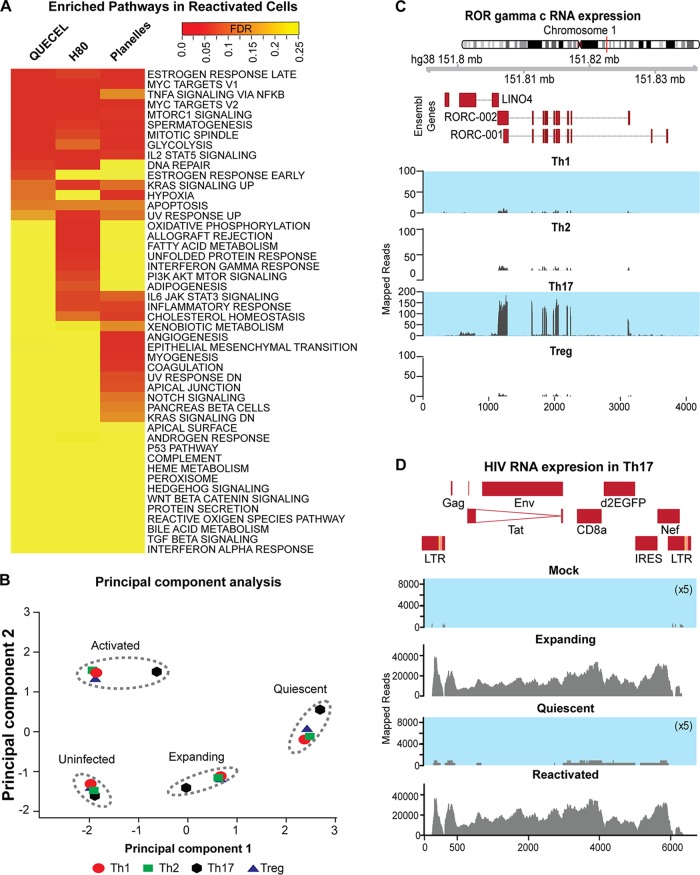
RNA-Seq analysis of cells at the effector, quiescent, and reactivated stages of the QUECEL method. (A) Pathway analysis indicates that pathways most enriched during reactivation in QUECEL (Th17 cells) are also induced in H80 (BioProject accession number PRJNA376596) and Planelles (BioProject accession number PRJNA322599) *ex vivo* primary cell models after reactivation. Genes differentially expressed following reactivation of quiescent cells were identified in all three data sets through pairwise comparison of RNA-Seq expression patterns of the quiescent versus reactivated cells and matched to the HALLMARK gene set of the mSigDB using the GSEA tool. The false discovery rate values were used to draw the heat map while controlling for the direction of enrichment. PI3K, phosphatitylinositol 3 kinase. (B) Principal component analysis of expanding, quiescent, and reactivated infected T helper cell subsets and expanding uninfected control using RNA-Seq data. (C) RORγC mRNA expression in each of the T helper cell subsets. Normalized read histograms of RNA-Seq data from uninfected cells are shown. The position of features in nucleotides on hg38 chromosome 1 and the position of the introns and exons of the two isoforms of RORC are shown at the top. (D) HIV mRNA expression in expanding, quiescent, and reactivated infected Th17 T cells and the uninfected control. For panels C and D, the scales to the left of the histograms represent normalized RNA-Seq reads. For uninfected expanding and infected quiescent cells, the scales have been 5× expanded to visualize the small number of mapped reads. The HIV construct used in this study is shown at the top.

Successful cell polarization was initially analyzed using quantitative PCR (qPCR) mRNA expression arrays (Fig. S2) and confirmed by comprehensive transcriptome sequencing (RNA-Seq) analyses (Fig. 2) of exponentially growing cells (day 6). We were able to detect abundant mRNA transcripts that distinguish each T cell subset, including IFNG and STAT4 production in the Th1 subset, IL-4 and GATA2 in the Th2 subset, IL-17A and RORC in the Th17 subset, and FOXP3 and IL-2 in the regulatory T cell subset (Fig. S2) (43).

10.1128/mBio.00337-19.2 FIG S2 **QUECEL T cell subsets express cell specific mRNAs.** (A) Heat maps showing relative qPCR expression levels of 10 T cell subset-specific mRNAs (top) and the corresponding RNA-Seq levels (bottom). (B) Correlation plot of polarized Th17s compared to peripheral Th17s and fresh total CD4 memory T cells using the RT^2^ Profiler PCR array human Th17 response array (330231; Qiagen) (left) or RT^2^ Profiler PCR array human T helper cell differentiation array (330231; Qiagen) (right). Download FIG S2, TIF file, 1.8 MB. Copyright © 2019 Dobrowolski et al. 2019 Dobrowolski et al. This content is distributed under the terms of the Creative Commons Attribution 4.0 International license.

To gain further insight into the overall pattern of gene expression among the four polarized cells in exponentially growing, quiescent, and reactivated states, we performed a principal-component analysis of RNA-Seq data sets (Fig. 2B). This objective analysis of the gene expression profiles clearly demonstrated the close similarity of each of the cell growth states independent of the cell polarization conditions. However, within each cluster, Th17 cells were the most distinct for each of the 4 activation/infection states (Fig. 2B).

The polarization phenotypes were further confirmed using flow cytometry to measure cytokine expression (Fig. S3A and B) and specific transcription factors (Fig. S3C) (44). As expected, the vast majority of cells expressed the expected transcription factors and cytokines that were appropriate for their polarization phenotypes: Th1 (Tbet, 80%), Th2 (GATA3, 95.6%), Th17 (RORC and Tbet, 97%), and Tregs (FOXP3, 61%). RORγC is an important transcription factor only expressed in Th17 cells, and this transcript is only found in the Th17 subset (Fig. 2C) (45).

10.1128/mBio.00337-19.3 FIG S3 **Polarized QUECEL T cells express subset-specific cytokines and transcription factors.** (A) Flow cytometry of subset-specific transcription factors in unstimulated memory T cells isolated from PBMCs. (B) Cytokine expression levels in all T cell subsets. (C) Transcription factor expression in the cells shown in panel B. Download FIG S3, TIF file, 3.3 MB. Copyright © 2019 Dobrowolski et al. 2019 Dobrowolski et al. This content is distributed under the terms of the Creative Commons Attribution 4.0 International license.

We also compared a qPCR Th17-specific inflammation transcription array of RNA from cells generated *in vitro* to patient-derived peripheral Th17 cells (41, 42, 46). The polarized Th17 cell transcripts correlated to peripheral Th17 with an *R*^2^ value of 0.98336 (Fig. S2B).

The RNA-Seq data sets also allowed us to measure the HIV transcripts in expanding, quiescent, and reactivated populations (Fig. 2D). Analysis of the pattern of RNA-Seq reads mapping to the infected HIV construct indicated that the expanding infected and reactivated populations contained high expression levels of transcripts for the entire HIV genome, but these were largely absent from the quiescent cell population (the scale has been expanded 5-fold to show the residual reads). As expected, uninfected controls showed no HIV transcripts.

### Efficient induction of quiescence by cytokines.

**FIG 3:**
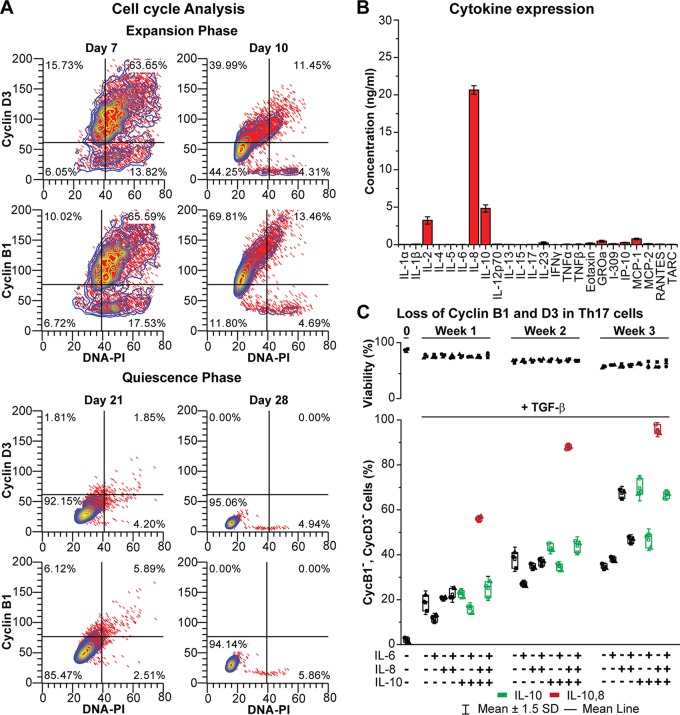
Induction of quiescence by cytokines. (A) Cell cycle analysis of cyclin D3, cyclin B1, and total DNA content (PI) during the expansion phase as well as the quiescence phase of Th17-polarized cells. (B) Cytokine secretion by H80 cells. (C) Loss of cell cycle markers in polarized Th17 cells exposed to different combinations of cytokines for 7 days and 10 ng/ml TGF-β.

To minimize the amount of cell death, subsets were gradually weaned of their expansion cytokines over a period of a week and then placed under reduced/protective cytokine conditions for 1 to 2 weeks. The cell cycle analyses shown in Fig. 3A documents that during this period, cells progressively lose cyclin B1 and D3 and enter the G_0_ phase (47).

Although infected polarized T cells will spontaneously senesce and drive HIV into latency (30, 35), this process was inefficient and highly variable in our experience. In our original primary cell model for HIV latency (29), we used H80 feeder cells (48) to gradually induce HIV silencing while promoting CD4^+^ T cell survival. Analysis of cytokine production by H80 cells showed that they overexpressed IL-2, IL-8, and IL-10 (Fig. 3B). A systematic analysis of the ability of the effector T-cells to enter quiescence when cultured for up to 4 weeks in combinations of IL-6, IL-8, and IL-10 in the presence and absence of TGF-β, a cytokine known to suppress activation of T cells (49, 50) is shown in Fig. 3C (with TGF-β) and Fig. S4A (without TGF-β). The combination of IL-8 and IL-10 strongly enhanced entry of cells into quiescence and latency, whereas IL-6 blocked entry into quiescence (Fig. 3C). Entry into quiescence was greatly enhanced by the addition of TGF-β, allowing the entire cell population to enter latency when cells are cultured for 3 weeks in the presence of TGF-β, IL-8, and IL-10 (Fig. 3C). There is a gradual loss of cells, due to senescence and apoptosis, throughout the shutdown process. Approximately 60% viable cells are recovered at 3 weeks, and there is no significant difference in cell viability in the presence of the different cytokine cocktails.

10.1128/mBio.00337-19.4 FIG S4 **The H13L Tat mutant does not affect reactivation of latent HIV.** (A) Cyclin B1 and D3 levels in Th17 cells treated with multiple cytokines in the absence of TGF-β. (B) Reactivation of latently infected Th17 cells carrying either WT, H13L Tat, or the inactivated C22G Tat mutant in response to a panel of activators. (C) Reactivation of latently infected Jurkat clones carrying either H13L Tat (2D10, G5) or WT Tat (E4). Download FIG S4, TIF file, 1.8 MB. Copyright © 2019 Dobrowolski et al. 2019 Dobrowolski et al. This content is distributed under the terms of the Creative Commons Attribution 4.0 International license.

Entry into latency is driven primarily by the quiescence of the host cells. Reporter viruses carrying the H13L and wild-type (WT) Tat genes showed identical shutdown and reactivation profiles in this system (Fig. S4B). Similarly, in the Jurkat system, the reactivation profile of the H13L Tat gene was indistinguishable from that of the wild type (Fig. S4C).

### Quiescent cells have a unique RNA transcript profile.

Pathway analysis performed on the RNA-Seq data sets demonstrated a decline in the expression of genes associated with expanding cells in the quiescent cell population. The expression of these pathways is restored in quiescent cells treated with TCR agonists (Fig. 2A and Fig. S5A). The differentially enriched pathways included those associated with cell division (G_2_-M checkpoint and mitotic spindle), global induction of transcription and translation (MYC targets and MTORC1 signaling), and induction of metabolic states associated with rapidly expanding T cells (glycolysis and hypoxia) (Fig. S6). All of the pathways enriched in the QUECEL model are also enriched in RNA-Seq data sets obtained from cells that have entered quiescence after culturing on H80 feed cells (51), or by the Bosque and Planelles method (52), and the three data sets are highly correlated (Fig. 2A and Fig. S5). However, additional pathways associated with senescence and innate immune responses also become activated in the H80 and Bosque and Planelles models (Fig. 2A and Fig. S5A).

10.1128/mBio.00337-19.5 FIG S5 **Differential gene expression pattern following reactivation in QUECEL versus two other *ex vivo* primary cell models.** (A) Table of pathways and gene sets enriched during transition from quiescence to reactivation in Th17 cells. A false discovery rate cutoff value of 0.1 was used to identify gene sets and pathways that were significantly enriched. (B) Genes induced following reactivation in QUECEL show significant overlap those upregulated in H80 and Planelles *ex vivo* models, with downregulated genes showing a smaller amount of overlap. (C) Pairwise scatterplots indicate a strong overall correlation of changes in gene expression pattern following reactivation between the three *ex vivo* primary cell models. In order to compare gene expression data obtained from similar populations of cells between the three datasets, all four polarized cells in the QUECEL method (Th1, Th2, Th17, and Treg) have been used in aggregate to generate the lists of QUECEL up- and downregulated genes in panels B and C. Download FIG S5, TIF file, 2.1 MB. Copyright © 2019 Dobrowolski et al. 2019 Dobrowolski et al. This content is distributed under the terms of the Creative Commons Attribution 4.0 International license.

10.1128/mBio.00337-19.6 FIG S6 **Heat maps of gene sets corresponding to the top enriched pathways after TCR stimulation of quiescent cells (24 h).** Data indicate the strong induction of genes involved in metabolic, transcriptional, and translational activation of cells. The values graphed in the heatmaps correspond to the differential expression value (in log_2_ units) obtained by pairwise comparison of quiescent versus TCR-stimulated cells. Note that while the shown pathways were originally found to be enriched using only the Th17 polarized cells, the panels in this figure have been generated using the aggregate of all four polarized cells in the QUECEL method (Th1, Th2, Th17, and Treg), indicating the reproducibility and generality of these results across the effector T cell subsets. Download FIG S6, TIF file, 2.4 MB. Copyright © 2019 Dobrowolski et al. 2019 Dobrowolski et al. This content is distributed under the terms of the Creative Commons Attribution 4.0 International license.

### Latently infected proviruses are restricted for transcription initiation and elongation.

To demonstrate RNA polymerase II (RNAP II) recruitment to the HIV long terminal repeats (LTRs) after TCR activation of the latently infected cells, chromatin immunoprecipitation (ChIP) assays were performed using primers that specifically amplified either the 5′ LTR, 3′ LTR, or the promoter region of glyceraldehyde-3-phosphate dehydrogenase (GAPDH) as a control (23) (Fig. S7). In contrast to Jurkat cells (23, 27, 53), initiation is highly restricted, and only a minimal level of paused, promoter-proximal RNAP II is seen at the LTR in the unstimulated cells. This can only be detected using an antibody against total RNAP II, suggesting that it is hypophosphorylated (54). Within 0.5 h of TCR stimulation there is a significant recruitment of pSer5 RNAP II at the 5′ LTR of HIV and a significant increase in both pSer5 RNAP II and pSer2 RNAP II at the 3′ LTR at the 16-h time point due full Tat-dependent elongation of the provirus (23). As an additional control, we also evaluated levels of H3K27me3, a methylated histone that is known to accumulate on latent proviruses and is removed during activation (28, 36). A high level of H3mK27me3 is present at the 5′ LTR of the unstimulated cells, and this is lost upon activation (Fig. S7B).

10.1128/mBio.00337-19.7 FIG S7 **Chromatin immunoprecipitation (ChIP) analysis of the latent provirus in Th17 cells.** (A) Location of primers used in the ChIP assays to specifically analyze the 5′ and 3′ HIV LTRs. (B) ChIP assays. Cells were stimulated through the TCR for 30 min or 16 h (*n* = 3). (Top) Total RNAPII; RNAP II (pSer5). (Bottom) RNAP II (pSer2) and methylated histone H3 (H3K27me3). GAPDH was used as a control. Download FIG S7, TIF file, 1.4 MB. Copyright © 2019 Dobrowolski et al. 2019 Dobrowolski et al. This content is distributed under the terms of the Creative Commons Attribution 4.0 International license.

### The QUECEL model accurately reflects the behavior of LRAs in patient cells.

Because the QUECEL model allows recovery of large homogeneous populations of latently infected cells, it is highly suitable for the screening of latency-reversing agents (LRAs). In addition to induction of Nef, we routinely monitor pSer175 CDK9, which is a marker of P-TEFb reactivation status (38). In quiescent cells, there are minimal levels of both HIV Nef and pSer175 CDK9 (<1%), and any increase in their levels is indicative of reactivation of the latent HIV provirus (Fig. 1C).

**FIG 4:**
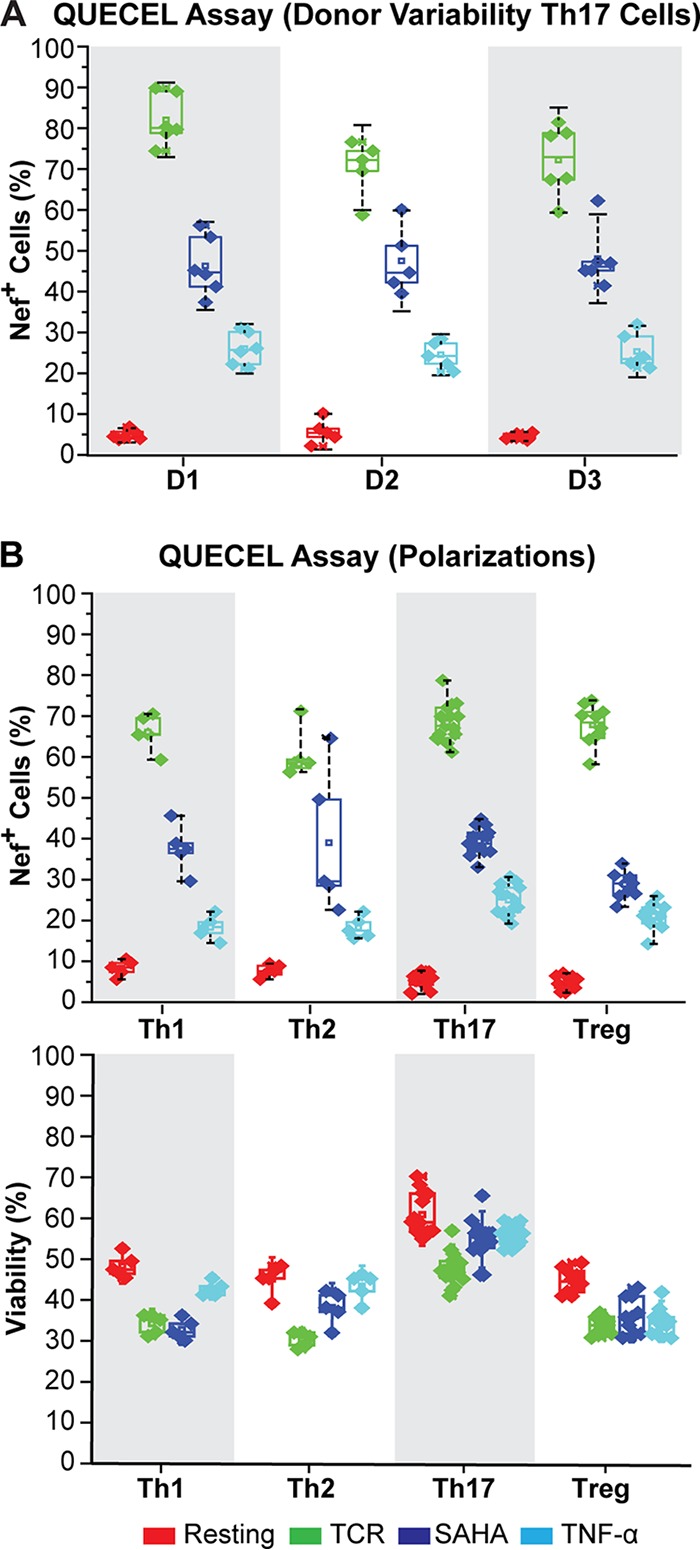
Reproducibility of the QUECEL model. (A) Donor variation (Th17 cells). Induction of HIV Nef expression in latently infected Th17 cells stimulated with TCR beads, SAHA, or TNF-α was measured in separate experiments using cells derived from three donors (donor 1, *n *= 7; donor 2, *n* = 6; donor 3, *n* = 6). (B) Induction of HIV Nef expression in each of 4 different T helper subsets stimulated with TCR, SAHA, and TNF-α (Th1, *n* = 4; Th2, *n* = 4; Th17, *n* = 10; Treg, *n* = 8). (Top) Cells were obtained from 3 different donors. (Bottom) Viability of cells measured by light scatter.

To determine the reproducibility of the system, we tracked HIV Nef expression between multiple donors (*n* = 3) over many separate experiments (*n* = 6) performed during the last 4 years. For each donor, cells were reactivated using either TCR, suberoylanilid hydroxamic acid (SAHA), or TNF-α, and the total amount of Nef expression was scored (Fig. 4A). The same stimulation conditions were also tested in all four T helper subsets over multiple experiments (*n* = 5) (Fig. 4B). The responses of the different subsets to the latency-reversing agents was virtually identical. However, we prefer to work with the Th17 cells, since they have the lowest basal activation levels and highest viability, most likely due to their reliance on IL-23, compared to IL-2 alone for the other T helper subsets (Fig. 4B).

**FIG 5:**
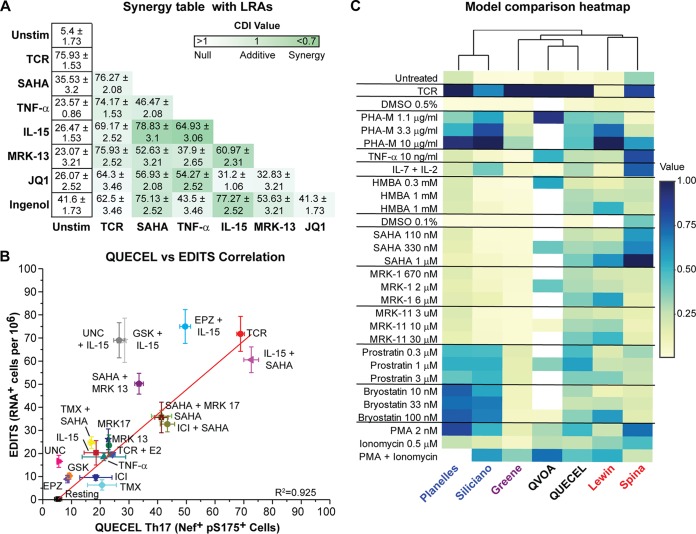
Correlation between the QUECEL model and RNA induction in patient cells. (A) Synergistic reactivation of HIV in Th17 cells (% Nef^+^, pS175 CDK9^+^ cells after 24 h). Green color intensity represents BLISS scores demonstrating additive or synergistic (below 1) activation. (B) Correlation between reactivation of HIV in patient cells (EDITS, vertical axis) and QUECEL model (Th17 cells, horizontal axis) for a wide number of activation conditions. Data were fitted with a linear model (*R*^2^ = 0.941; *n* = 18). (C) Heatmap of responses of QUECEL (Th17 cells) to a panel of primary cell latency models analyzed by Spina et al. (26). Note that the QUECEL model clusters mostly with the QVOA and has a broader dynamic range than the other primary cell models of latency. Normalized values were calculated relative to the highest activation within each model and expressed as a percentage of maximal stimulation. DMSO, dimethyl sulfoxide. PHA-M, phytohemagglutinin-M; PMA, phorbol myristate acetate.

When LRAs are combined, synergistic activation conditions can be demonstrated (Fig. 5A). It is especially noteworthy that a combination of IL-15 and SAHA resulted in activation levels that are statistically indistinguishable from those of TCR stimulation (Fig. S8B). The amount of synergism was measured by the coefficient of drug interaction (CDI), where a value of 1 is considered additivity and anything below 0.7 is considered synergistic. IL-15 synergized best with SAHA, followed by TNF-α and Ingenol (Fig. 5A).

10.1128/mBio.00337-19.8 FIG S8 **Synergistic proviral reactivation in the QUECEL assay.** (A) Representative flow plots of HIV Nef^+^ and pS175 CDK9^+^ Th17 cells treated with IL-15 (10 ng/ml) alone or in combination with SAHA (500 nM) and TNF-α (10 ng/ml). (B) Correlation between the QUECEL (Th17) model (top, *n* = 6) and RNA induction in patient cells (EDITS assay, bottom, *n* = 10). (C) HIV reactivation in Th17 cells treated with IL-15 in combination with other LRAs (*n* = 3). (D) Heat map comparison of QUECEL and QVOA reactivation to latently infected Jurkat cell clones from the Karn laboratory (2D10, E4, and G5) and the Verdin laboratory (JLat 5A8, JLat 8.4, JLat 6.3, and JLat 11.1). Values were normalized compared to highest stimuli within each model and expressed as percentage of maximal stimulation. Download FIG S8, TIF file, 2.0 MB. Copyright © 2019 Dobrowolski et al. 2019 Dobrowolski et al. This content is distributed under the terms of the Creative Commons Attribution 4.0 International license.

The QUECEL results were compared to the activation profiles of well-suppressed HIV-infected patient CD4 memory cells (*n* = 11) using the EDITS assay (55) (Fig. 5B). The hierarchy of responses was comparable for a wide range of different activators, and the data were highly correlated. However, basal activation levels are higher in the QUECEL system than in the patient cells, and the patient cells are generally more restricted in their responses to LRAs. Notably, the synergy between IL-15 and SAHA is maintained, and latency reversal under these conditions is comparable to that seen after TCR activation.

HIV reactivation in the QUECEL model was also compared to that of a wide variety of previously tested latency models using the same reactivation conditions, as described by Spina et al. (26) (Fig. 5C). It is notable from the heat map that the QUECEL data clustered most closely with the quantitative viral outgrowth assay (QVOA) data (Fig. 5C) and showed the broadest range of responses to the various activators, including hexamethylene bisacetamide (HMBA) and SAHA. As expected, there is a poor correlation between the responses of Jurkat cells (E4 [wild-type Tat], 2D10 [H13L Tat], and G5 [H13L Tat and Nef]) and the QUECEL model (Fig. S8D) due to the dependence of Jurkat cells on NF-κB for HIV transcription (17) and their comparatively poor responses to TCR stimulation (22).

### HIV reactivation in primary cells is dependent primarily upon NFAT.

The small-molecule inhibitors cyclosporine and the IKK inhibitor IV, which block activation of NFAT and IKK, respectively, were used to assess the relative contributions of NFAT and NF-κB to proviral reactivation in the QUECEL model (Fig. S9). The cells were pretreated with these compounds for 2 h and then subsequently stimulated with TCR, TNF-α, or SAHA. An Amnis ImageStream analysis of the nuclear localization of NF-κB and NFATc1 after stimulation with LRAs confirmed that there was a significant increase in both NF-κB (50%) and NFAT (68%) after TCR stimulation but no significant activation of these factors by SAHA (Fig. S9A). Inhibition of NFAT induction by cyclosporine resulted in an 8-fold decrease in Nef and pS175 CDK9 double-positive cells after TCR stimulation and a 3-fold decrease after SAHA stimulation (Fig. S9B and C). However, there was no inhibition of the TNF-α-treated cells. In contrast, blocking of NF-κB had a marginal effect on TCR and SAHA stimulation but a 6-fold effect on TNF-α stimulation. TNF-α resulted in a significant increase in NF-κβ recruitment to the nuclei, even though there was limited HIV Nef expression under these conditions. Thus, in sharp contrast to the Jurkat cell system, there is relatively low contribution of NF-κB to proviral reactivation compared to that of NFAT (30, 56, 57).

10.1128/mBio.00337-19.9 FIG S9 **NFAT is the dominant transcription initiation factor regulating HIV latency in the QUECEL model.** (A) Amnis ImageStream analysis of NFκB-p65 and NFATC1 localization in quiescent Th17 cells treated with LRAs. (B) HIV reactivation in quiescence stimulated through the T-cell receptor in the presence of cyclosporin (CsA) (1 µM) or IKK (1 µM). (Top) Resting cells. (Bottom) TCR-treated cells (24 h). (C) HIV reactivation in Th17 cells treated with CsA and IKK prior to activation with TCR, SAHA, and TNF-α (*n* = 3). Download FIG S9, TIF file, 3.5 MB. Copyright © 2019 Dobrowolski et al. 2019 Dobrowolski et al. This content is distributed under the terms of the Creative Commons Attribution 4.0 International license.

## DISCUSSION

### Entry into quiescence forces HIV into latency.

The most likely explanation for the generation of latently infected cells in patients is that HIV-1 initiates infection in activated CD4^+^ T cells, which are highly permissive for infection and support active viral gene transcription, but the virus then becomes silenced as host cells transition to a quiescent (resting) state (13, 14). In the QUECEL model, we recapitulate the transition from effector cells to memory cells under highly defined conditions.

One key innovation of the QUECEL model is the use of effector cells polarized into each of the major helper subsets. Helper cells with a Th17 (58) or Th2 (59) phenotype specialize in antifungal, antihelminthic, and antitumor immune defense, while Th1-polarized cells are predominantly directed against viral pathogens (51). The Lichterfeld group (52) has recently demonstrated that clonal proliferation of replication-competent virus can be observed in each of these polarization subsets but especially in cells with a Th1 or Th17 phenotype. The Th17 polarization phenotype, in which we have successfully modeled HIV latency for the first time, represents the most abundant effector T cell population in the lamina propria of the gastrointestinal tract (60, 61) and a target for HIV infection (62).

We have rigorously characterized each of these polarization states by flow cytometry assays of transcription factor and cytokine expression and comprehensive RNA-Seq analyses. Although each phenotype is distinct, comparison of the RNA-Seq data sets obtained from the QUECEL and two related primary cell models of HIV latency demonstrate clearly that the dominant driver of HIV latency is entry of cells into quiescence (63–66). However, in contrast to White et al. (66), we did not see enrichment for the p53 pathway in the data set. Instead, the dominant factors controlling entry into quiescence appear to be associated with c-myc-dependent pathways. Nonetheless, there are subtle differences in the utilization of signaling pathways between the different polarized cell types, and this may influence the responses of HIV to exogenous stimulation.

Previous investigators have relied on cellular exhaustion and/or relaxation for cells to undergo the transition to a quiescent state (13, 29, 30, 33, 35, 48, 67). In our initial primary cell model for HIV latency, cells were cocultured with H80 cells to permit them to gradually enter quiescence (29, 48, 63). However, this process took many weeks and was variable. In an effort to develop more defined conditions to force cells into quiescence, we analyzed cytokine production by H80 cells and found that they overproduced IL-10 and IL-8. Although these cytokines inefficiently promoted entry of cells into quiescence, they were synergistic with TGF-β, and this combination allowed us to force cells into quiescence. To confirm that the cells are fully quiescent, we routinely performed cell cycle analysis experiments, which show absence of cell cycle-dependent cyclins D3 and B1 and severe restrictions on the expression of CD69, CD25, and P-TEFb.

The main function of IL-10 is to induce an anti-inflammatory response. IL-10 is elevated during HIV, hepatitis C virus, and hepatitis B virus infections (68). The IL-10/IL-10R pathway is a key regulator of viral persistence, and blockade of IL-10R by anti-IL-10 monoclonal antibodies clears the chronic lymphocytic choriomeningitis virus (LCMV) infection in a mouse model (69). TGF-β is another anti-inflammatory/profibrotic cytokine which remains persistently elevated in both untreated and virally suppressed HIV-infected persons (70). The antiproliferative effects of TGF-β on TCR-activated cells is well documented (71, 72). TGF-β also inhibits IL-7-induced proliferation in memory, but not in naïve, human CD4^+^ T cells by interfering with c-myc induction (73, 74). Notably, in our model, c-myc pathways are highly repressed in the quiescent cells. IL-8, a CXC chemokine, which is also raised in the peripheral circulation of HIV-1-infected individuals (75), also interferes with HIV replication and suppresses HIV induction in the U1 cell model of HIV latency (76, 77). The fact that all these cytokines are elevated in HIV-infected individuals suggests that they play a role in maintaining and reseeding the HIV reservoir.

### The QUECEL model accurately recapitulates the transcriptional behavior of patient cells.

There is an excellent correlation between a wide range of latency-reversing agents in the QUECEL model and RNA induction in highly active antiretroviral therapy-treated, well-suppressed HIV patient cells, as measured by the EDITS assay. This was true not only for the very potent activators, such as TCR stimulation, but also for less potent activators, such as SAHA, and synergistic agents, such as SAHA used in combination with IL-15.

In contrast, the responses of the Jurkat cell model system to latency-reversing agents generally shows a poor correlation to RNA induction in patient cells. One key molecular difference is that P-TEFb is constitutively expressed in Jurkat T cells, whereas induction of P-TEFb plays a crucial role in the reactivation of HIV latency in primary cells (39). Reactivation of cells in the QUECEL model is strictly correlated with reactivation of PTEF-b and phosphorylation of pSer175 on CDK9, which is a prerequisite for efficient P-TEFb binding to Tat (38, 39). In contrast to Jurkat cells, which are highly dependent upon NF-κB activation (27, 53), reactivation in the QUECEL model is primarily dependent upon NFAT activation and NF-κB is largely dispensable.

Because of highly variable protocols, there is also a poor correlation between different primary cell latency models and how closely they mimic what is occurring in the patient samples (26). For example, TNF-α and SAHA were ineffective in the Bosque and Planelles model, but both are partially effective in the QUECEL model and patient cells.

In summary, we have developed methods to establish HIV latency in primary effector cells forced to enter quiescence by exposure to a defined cocktail of cytokines. The QUECEL model gives highly reproducible results using multiple donors and many different experiments. Since homogeneous populations of latently infected cells can be recovered, there is an excellent signal-to-noise ratio and no need to normalize for variations in infection efficiency. We therefore believe the QUECEL model will become an invaluable tool to study the molecular mechanisms underlying HIV latency.

## MATERIALS AND METHODS

### Polarization of CD4 T cell subsets.

A detailed protocol is provided in the supplemental material. Briefly, naive CD4 T cells were isolated using a RoboSep CD4 Naïve T cell negative selection kit (Stemcell). Naive CD4 T cells (2 × 10^6^) were resuspended in 10 ml RPMI medium and stimulated with 10 µg/ml concanavalin A (ConA) (EMD Milipore) in the presence of subset-specific cytokines (Table 1). Cells were cultured for 72 h at 37°C, followed by addition of 10 ml of fresh medium, additional 10 µg/ml ConA, polarization cocktail cytokines, and 120 IU/ml of IL-2. After 6 days, the cells were washed and placed into primary cell RPMI medium with growth cytokines of IL-23 (50 ng/ml) and IL-2 (60 IU/ml) for Th17 cells and IL-2 (60 IU/ml) for Th1, Th2, and Treg cells.

### Infection of polarized CD4^+^ T cells.

The polarized CD4 T cells were infected at a multiplicity of infection (MOI) of 2.0 at 5 × 10^6^ cells per ml using VSV glycoprotein-pseudotyped virus (22), in the presence of cell subset cytokines, in a 24-well plate. Cells were spinoculated at 2,000 × *g* for 1.5 h at room temperature and then placed in an incubator overnight. The cells were adjusted to 1 × 10^6^ per ml in the presence of cell subset-appropriate growth cytokines. After 48 h, infection efficiency was determined by GFP expression.

### Isolation of HIV-infected cells using magnetic isolation technology.

After transduction, HIV-infected cells were isolated using RoboSep mouse CD8a positive selection kit II (Stemcell). Cells (50 × 10^6^ per ml) were preincubated with 50 µl/ml of antibody cocktail and 40 µl/ml of magnetic beads and diluted into 2.5 ml RoboSep buffer. The positive cells were recovered by magnetic bead separation, suspended in 1 ml of medium, and vortexed to release the cells and beads from the tube wall. Positively selected cells were placed in primary cell RPMI medium with cell type-specific growth cytokines at 1 × 10^6^ in upright flasks and allowed to expand for a week.

### Quiescence of polarized CD4 T cell subsets.

The number of cytokines was slowly reduced by incubating cells for 1 week without medium changes. Cell were resuspended in IL-2 (15 IU/ml), IL-23 (12.5 ng/ml), TGF-β (10 ng/ml), IL-10 (10 ng/ml), and IL-8 (50 ng/ml) for Th17 cells or IL-2 (15 IU/ml), TGF-β (10 ng/ml), and IL-8 (50 ng/ml) for Th1, Th2, and Treg cells. Entry of cells into quiescence was measured by the reduction in cyclin D3, pSer175 CDK9, and HIV Nef levels. This takes anywhere from one to two additional weeks depending on donor and experimental variation.

### Stimulation procedure and inhibitor panel.

Quiescent cells (2 × 10^5^) were stimulated for 24 to 48 h in a U-bottom 96-well plate in the presence of either 1 TCR Dynabead per cell (Life Technologies), 500 nM SAHA (Caymen Chemical), 10 ng/ml TNF-α, 10 ng/ml IL-15, or 5 ng/ml IL-7 (all from Peprotech). For the transcription inhibitor panel, 2 × 10^5^ cells were pretreated for 2 h prior to stimulation with one of the following: 1 µM cyclosporine (Sigma-Aldrich), 1 µM Go6976 (Sigma-Aldrich), or 1 µM IKK inhibitor IV (Santa Cruz Biotechnologies).

### Partially automated chromatin immunoprecipitation.

Quiescent Th17 cells were stimulated either for 30 min or overnight using Dynabeads in a 96-well U-bottom plate. ChIP was performed on 1 × 10^6^ cells using our previously described partially automated ChIP protocol (23). Antibodies used for immunoprecipitations were anti-RNAP II (sc-899; Santa Cruz Biotechnologies), anti-RNAP II pSer5 (ab5131; Abcam), anti-RNAP II pSer2 (ab5095; Abcam), and anti-H3K27me3 (17-622; Millipore). DNA was amplified using HIV-specific primers (see Fig. S7 in the supplemental material), and each sample was barcoded for Ion Torrent sequencing with Ioncode barcodes. Sequences were separated by barcode, trimmed, and mapped to a pHR′-Nef-GFP/CD8a sequence.

### EDITS analysis from HIV-infected individuals.

Well-suppressed cART-treated patient peripheral blood mononuclear cells (PBMCs), from a UCSF patient cohort, were tested using the EDITS assay as previously described (55).

### Flow cytometry.

Samples were fixed in 4% formaldehyde (Electron Microscope Sciences) for 15 min and permeabilized with a Saponin-based buffer (Cytofix/Cytoperm; BD Biosciences) for 5 min. An antibody cocktail containing AF647-HIV EH1 Nef (Jim Hoxie laboratory), AF750-pSer175 CDK9 (generated by Covance for our laboratory), and tetramethyl rhodamine isothiocyanate-cyclin D3 (Santa Cruz Biotechnologies) was added to each sample for 20 min. Samples were washed twice and run on a BD LSR Fortessa instrument. Flow cytometry analysis was performed using Winlist 9.0.

### RNA-Seq library preparation and analysis.

Total cellular RNA was extracted from Th1-, Th2-, Treg-, and Th17-polarized exponentially growing cells (day 6) prior to infection with the HIV construct and 72 h postinfection. Additional samples were taken for each polarized cell population after the full induction of quiescence and HIV latency (day 28) and 24 h after TCR agonist-mediated stimulation of quiescent cells to induce reactivation. After RNA samples from all time points were taken, library preparation was performed using the TruSeq RNA sample preparation and single-end cluster generation kits from Illumina, which include a poly(A)+ enrichment step. ERCC Exfold RNA spike-ins (Life Technologies) were added according to the manufacturer’s instructions. High-throughput sequencing was performed on an Illumina HiSeq2000 instrument.

### Data availability.

The RNA-Seq data sets, comprising two replicate studies performed over a year apart, have been submitted to the SRA database (accession number SRP145508). All RNA-Seq data sets passed the quality control step, which was performed using FastQC (Babraham Bioinformatics).

10.1128/mBio.00337-19.10 TEXT S1 Detailed protocol. Download Text S1, PDF file, 0.3 MB. Copyright © 2019 Dobrowolski et al. 2019 Dobrowolski et al. This content is distributed under the terms of the Creative Commons Attribution 4.0 International license.
