# Supplementary figures and images for: Entry of Polarized Effector Cells into Quiescence Forces HIV Latency
Source: mBio. 2019 Mar 26;10(2):e00337-19. doi: 10.1128/mBio.00337-19 (PMC6437053; doi:10.1128/mBio.00337-19)

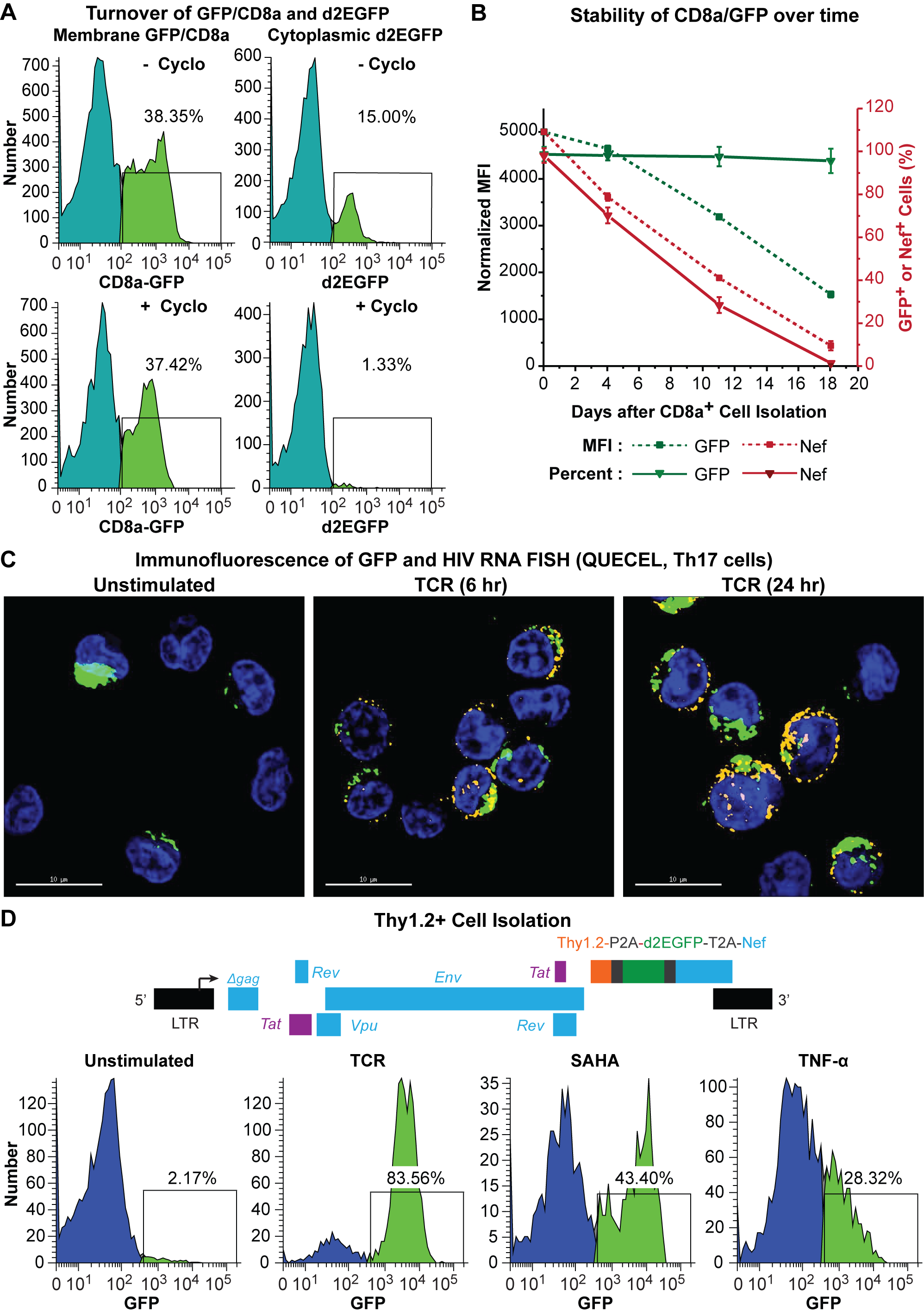

Supplement: FIG S1 [file mBio.00337-19-sf001.tif]

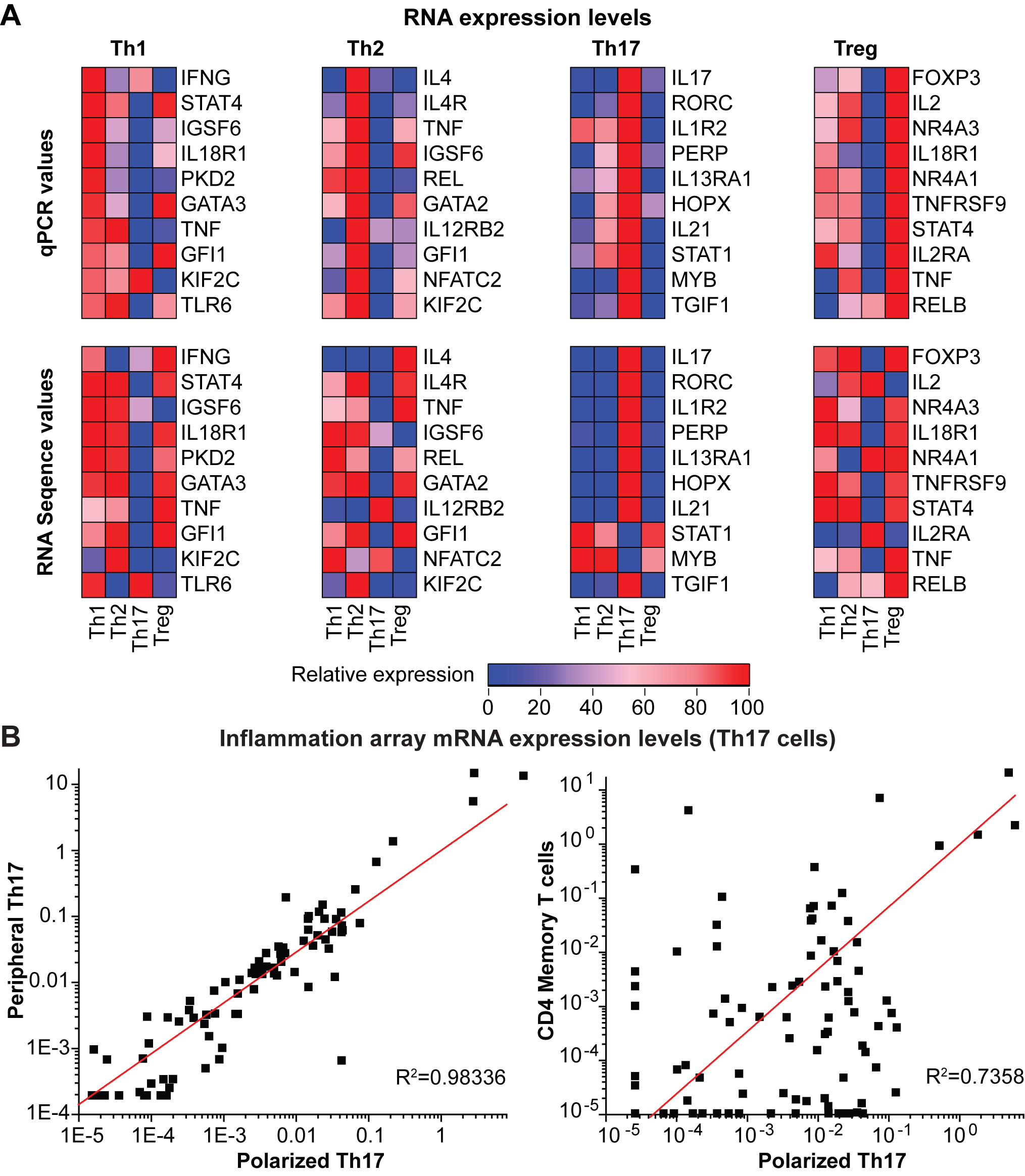

Supplement: FIG S2 [file mBio.00337-19-sf002.tif]

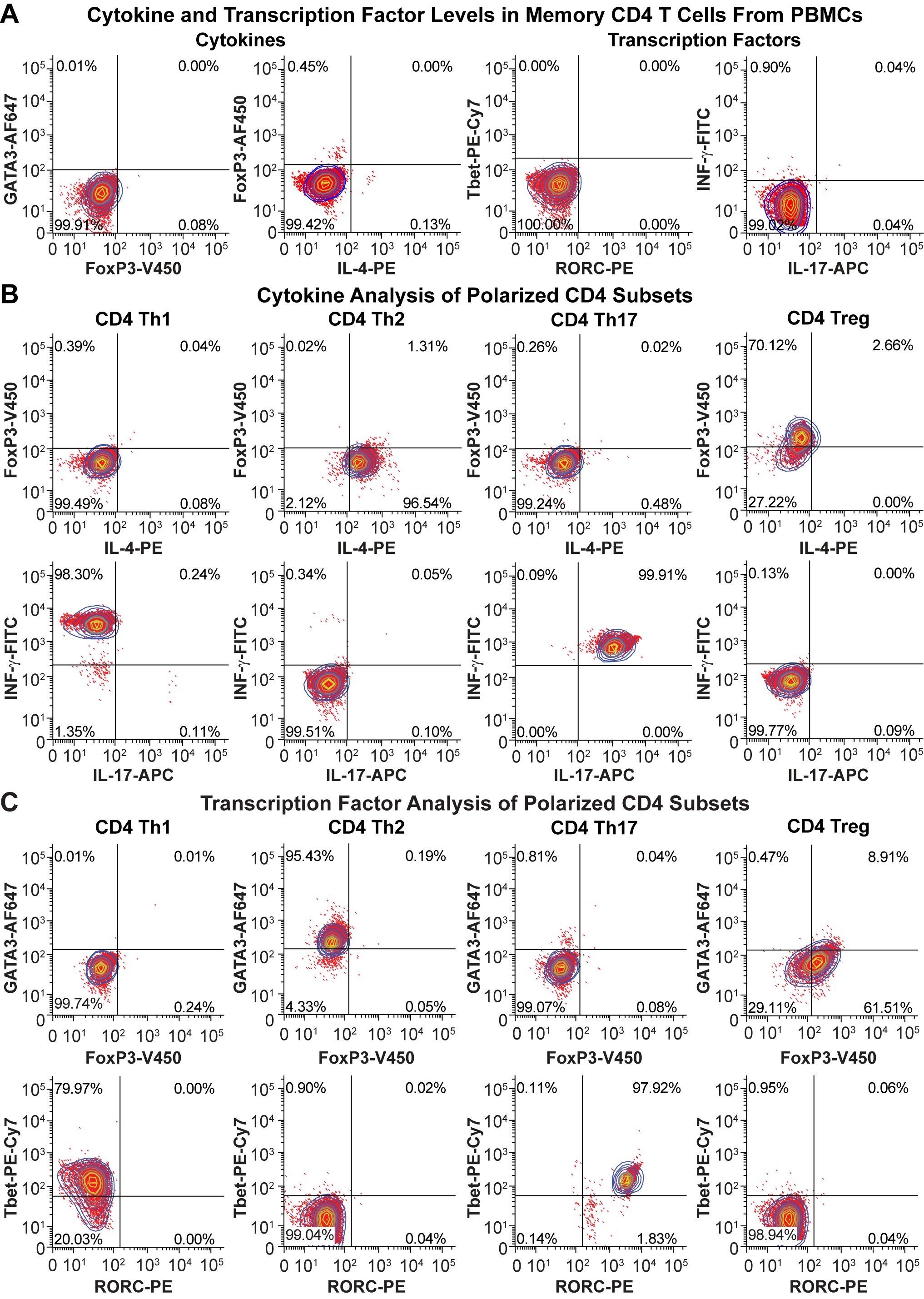

Supplement: FIG S3 [file mBio.00337-19-sf003.tif]

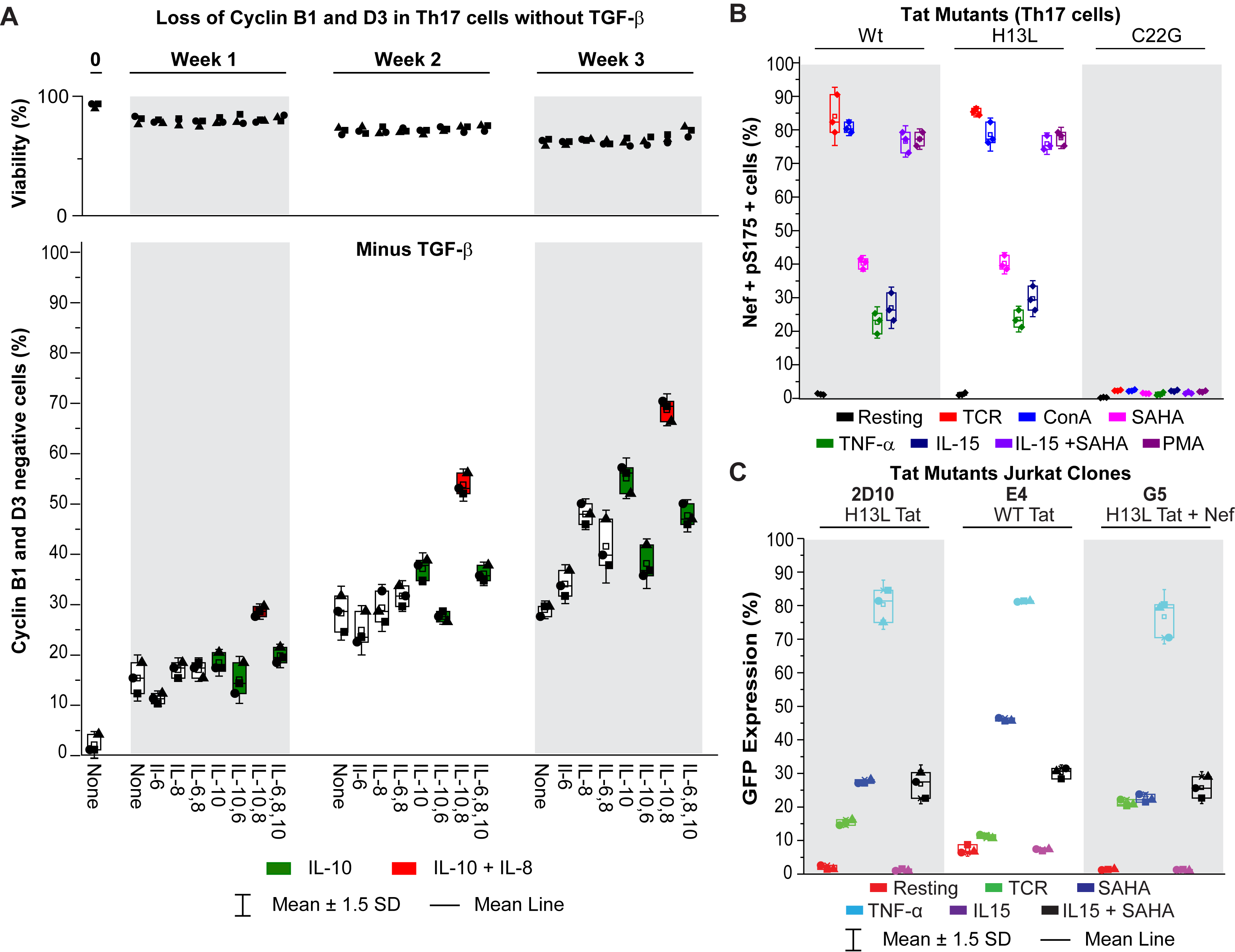

Supplement: FIG S4 [file mBio.00337-19-sf004.tif]

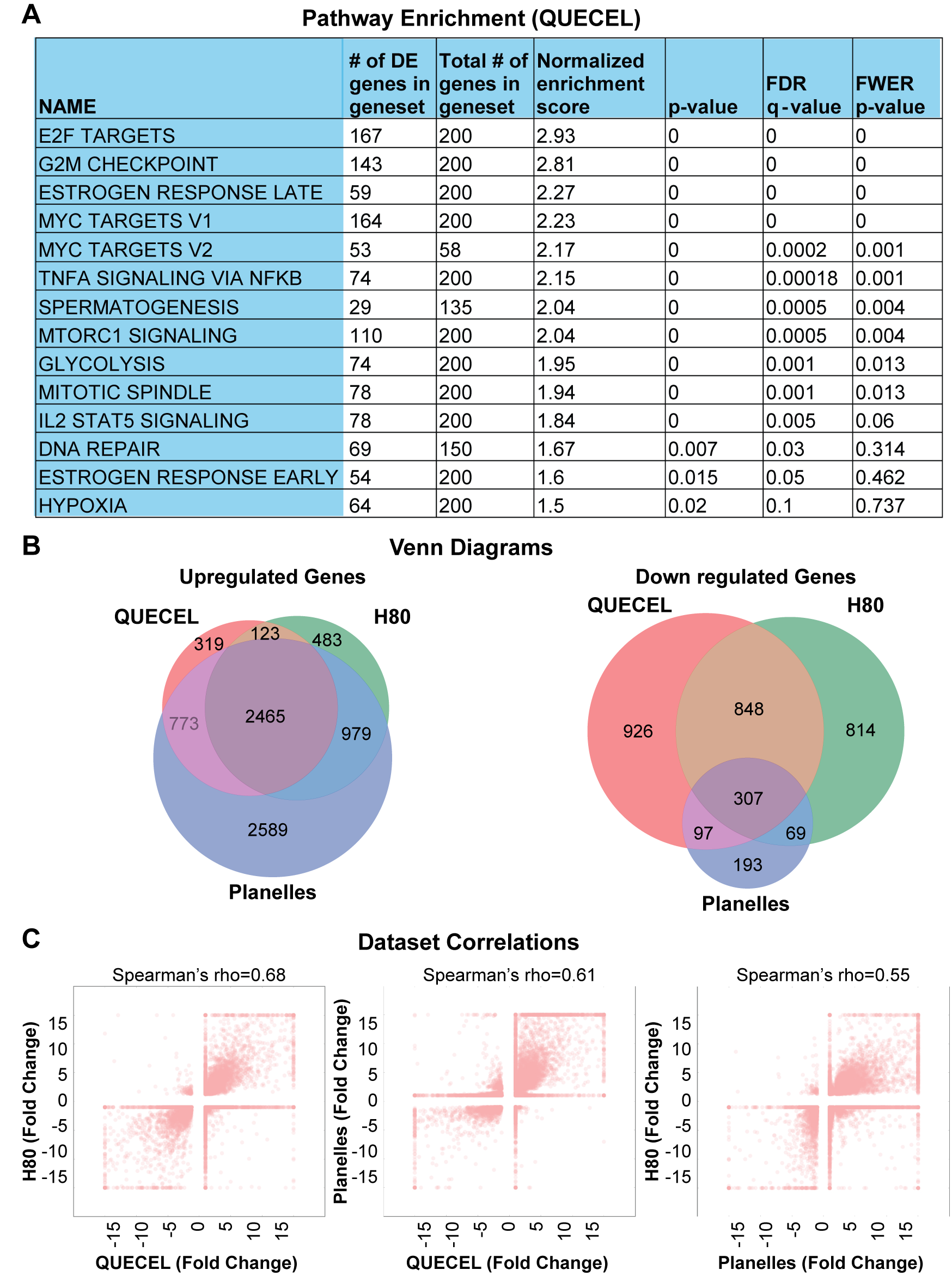

Supplement: FIG S5 [file mBio.00337-19-sf005.tif]

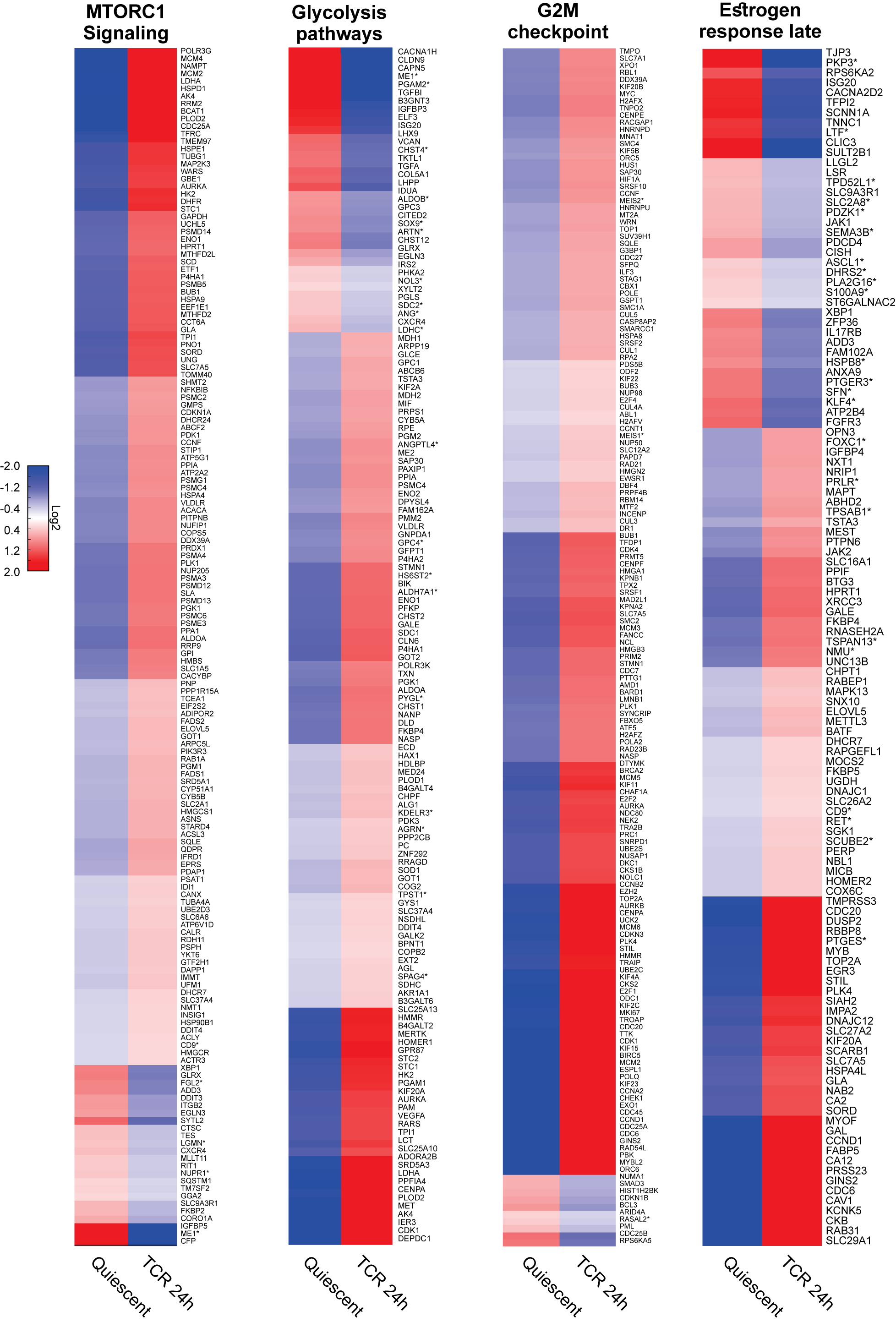

Supplement: FIG S6 [file mBio.00337-19-sf006.tif]

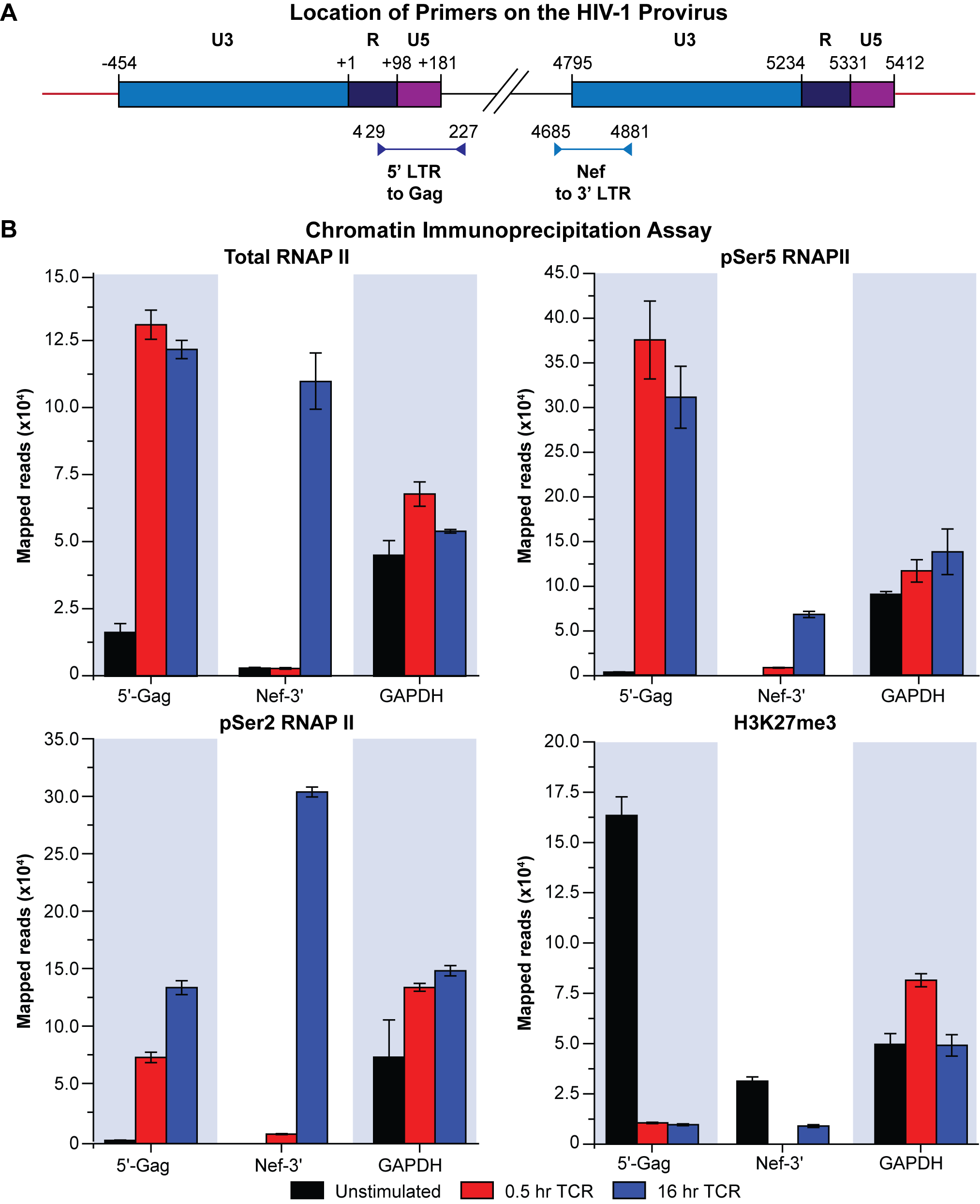

Supplement: FIG S7 [file mBio.00337-19-sf007.tif]

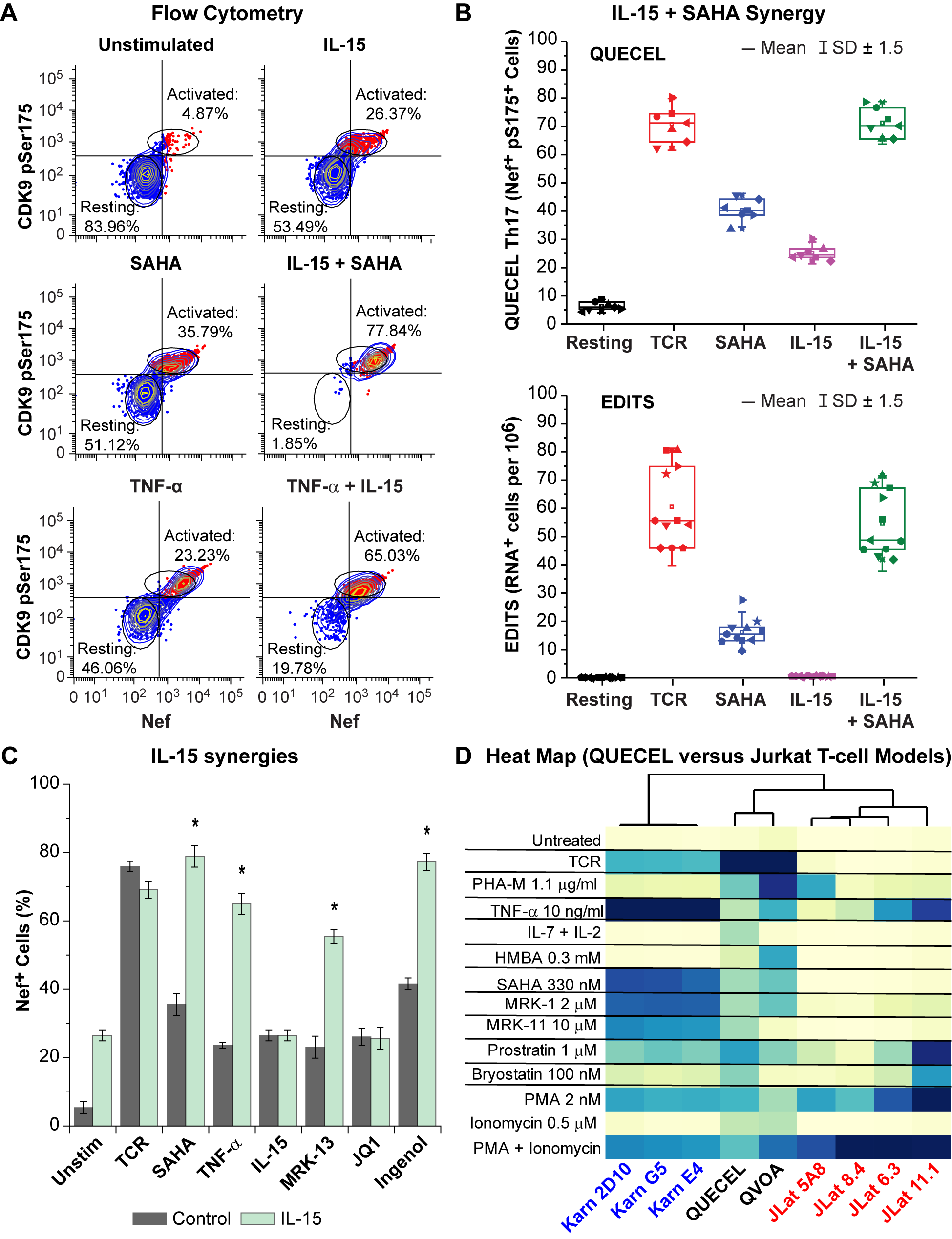

Supplement: FIG S8 [file mBio.00337-19-sf008.tif]

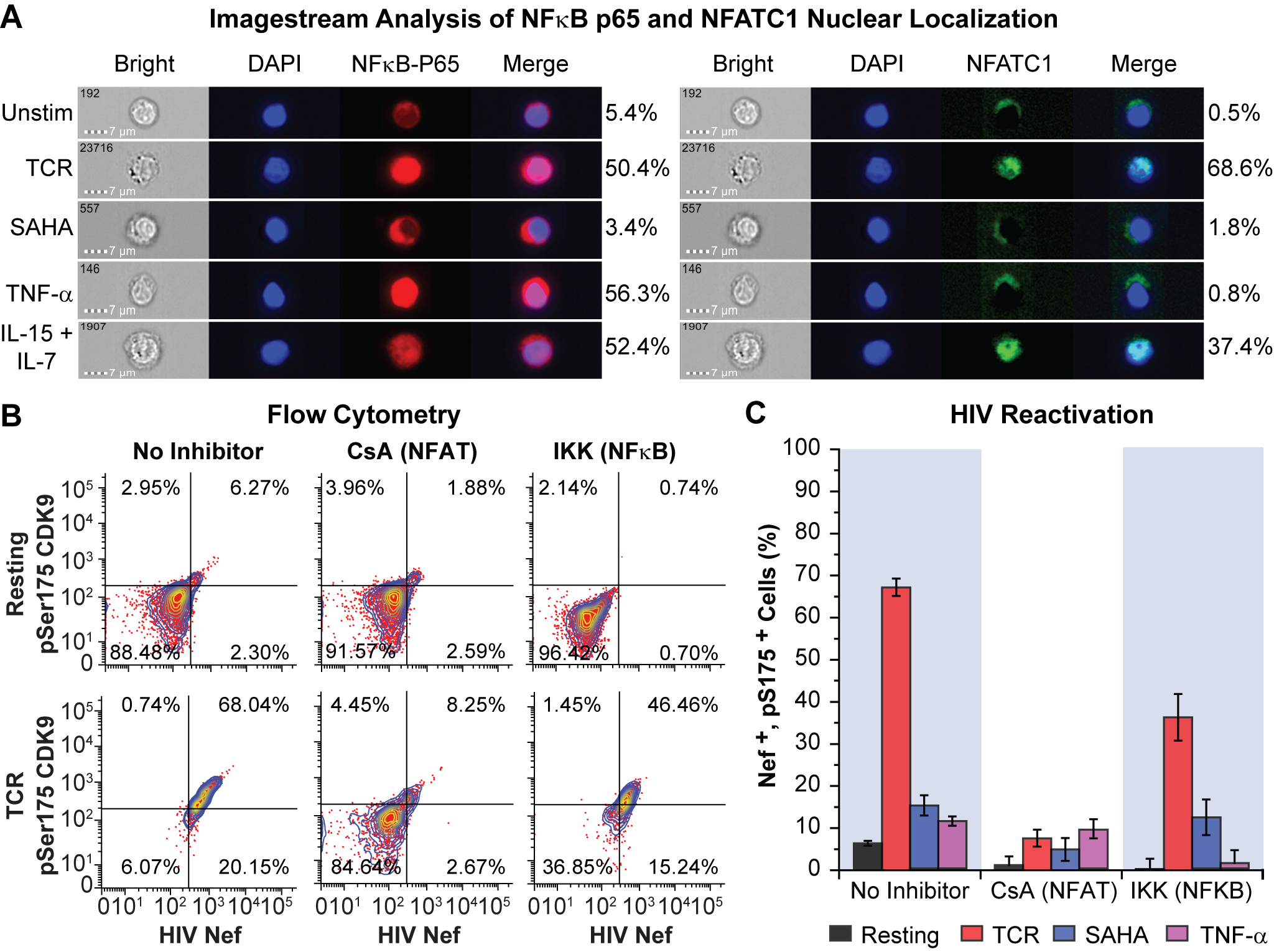

Supplement: FIG S9 [file mBio.00337-19-sf009.tif]
