# Supplementary material for: Entry of Polarized Effector Cells into Quiescence Forces HIV Latency
Source: mBio. 2019 Mar 26;10(2):e00337-19. doi: 10.1128/mBio.00337-19 (PMC6437053; doi:10.1128/mBio.00337-19)
Supplement: TEXT S1 [file mBio.00337-19-s0001.pdf]

QUECEL Protocol

General Passaging and Maintenance

These cells need to be grown in primary cell media (RPMI, 10% FBS, Primocin and 25 mM HEPES) together with their required cytokines (**Table 1**). Unless stated otherwise the cells must remain in these cytokines at the specified concentration. These cells need to be grown at high density due to autocrine production of cytokines. A concentration of 1 to 3 x 10^6 per ml is ideal, and the cells will stop growing at a concentration below 1 x 10^6 per ml. It is preferable not to remove any old media from these cells, and simply add fresh media and cytokines to the cells to bring them up to the ideal concentration once the media begins to turn yellow.

Table 1: Maintenance Cytokines

Subtype	Cytokine	Stock Conc.	Dilution	Source	Catalog No.
Th1, Th2, TReg	rIL-2	60,000 IU/ml	1:1000	NIH AIDS Reagent Program	136
Th17	rIL-2<br>rIL-23	60,000 IU/ml<br>50 µg/ml	1:1000<br>1:1000	NIH AIDS Reagent Program<br>PeproTech	136<br>200-23

Day 0: Activation and Initial Polarization

1. Isolate naïve CD4 T cells using EasySep™ Human Naïve CD4+ T Cell Enrichment Kit (Stem Cell Technologies, Catalog No. 19155F) and the RoboSep™ Fully Automated Cell Separator (Catalog # 21000, 20119, 20155) following the manufacturer's directions. Typically, 50 x 10^6 PBMCs yields 5 x 10^6 naïve CD4 T cells. Resuspend the naïve CD4 T cells at 5 x 10^5 cells per ml, which is approximately 10 ml of media per 50 x 10^6 PBMCs used. 5 x 10^6 of polarized cells yields 100 to 150 x 10^6 effector cells after the expansion phase of the protocol.
2. Add polarization cytokines and antibodies (**Table 2**), together with Concanavalin A (Millipore Sigma, 234567-1GM) at a concentration of 10ug/ml final concentration µl per 10^6 naïve cells.
3. Distribute cells into a upright T25 flask to allow for cell to cell contact.
4. Incubate cells for 72 hours at 37 °C in a CO_2 incubator.

Table 2: Polarization Cytokines

Subtype	Cytokine or Antibody	Stock Conc.	Dilution	Source	Catalog No.
Th1	Anti-Human IFN-γ<br>Anti-Human IL-4	10 µg/ml<br>500 µg/ml	1:1000<br>1:1000	Preprotech<br>Preprotech	500-M90<br>500-M04
Th2	Anti-Human IL-4<br>Anti-Human IFN-γ	500 µg/ml<br>10 µg/ml	1:1000<br>1:1000	Preprotech<br>Preprotech	500-M04<br>500-M90
Th17	TGF-β<br>Anti-Human IL-4<br>Anti-Human IFN-γ<br>IL-1β<br>IL-6<br>IL-23	5 µg/ml<br>500 µg/ml<br>10 µg/ml<br>10 µg/ml<br>30 µg/ml<br>50 µg/ml	1:1000<br>1:1000<br>1:1000<br>1:1000<br>1:1000<br>1:1000	Preprotech<br>Preprotech<br>Preprotech<br>Preprotech<br>Preprotech<br>Preprotech	100-21C<br>500-M04<br>500-M90<br>200-01B<br>200-06<br>200-23
TReg	TGF-β<br>Anti-Human IL-4<br>Anti-Human IFN-γ<br>Anti-Human IL-12	5 µg/ml<br>500 µg/ml<br>10 µg/ml<br>500 µg/ml	1:1000<br>1:1000<br>1:1000<br>1:1000	Preprotech<br>Preprotech<br>Preprotech<br>Preprotech	100-21C<br>500-M04<br>500-M90<br>500-M12

Day 3: Continuing Polarization

5. Measure out the same amount of primary cell media used at day 0, and add polarization cytokines following the dilution **Table 2,** together with the same amount of Concanavalin A from step 2 and 120 IU/ml of IL-2 and add to the polarizing cells.
6. Incubate cells for 72 hours at 37 °C in a CO_2 incubator.

Day 6: Infection by lentiviral vector

Cells are ready for infection after the 6th day of polarization. Since primary cells are notoriously hard to infect the most efficient infections are achieved by spinoculation for a long period of time using a high titer virus stock as follows:

7. Pellet the polarized cells and discard the supernatant.
8. Use the pHR'-Nef+-CD8a/GFP virus when using the CD8a protein for purification of the infected cells. Use 1 ml of a high titer concentrated virus stock (MOI of 10 on Jurkat cells) per 10 x 10^6 cells.
9. Dilute the virus in sufficient primary cell media and cell-specific cytokines (**Table 1**) to resuspend the cells at 5 x 10^6 cells per ml (i.e. for 10 x 10^6 cells, add 1 ml primary cell media to 1 ml concentrated virus and 2 µl of cytokine stock).
10. Aliquot 1 ml of cells into each well of a 24 well plate. It is convenient to split the cells evenly between into two plates to provide a balance for the centrifugation.
11. Spin the plates at 2000 RCF for 90 min 23 °C and 5 acceleration/9 deceleration.

12. Remove the cells from the centrifuge and place at 37 °C in a CO_2 incubator overnight. This will increase the amount of infection.

Day 7: Dilution of Virus

13. Cells should have grown in number after the overnight incubation. Pool the cells and replate at 1 x 10^6 cells per ml. Do not remove the virus and simply dilute the cells with fresh media until cells are at the desired concentration.

Days 8-13: Cell Husbandry and Expansion

The cells were kept growing by addition of normal cytokines and fresh media once the media turns yellow (or after 3 days). Cell densities were maintained above 1 x10^6 million cells per ml until the needed amount of cells was reached. Check the amount of GFP^+ cells 48 hrs after infection and use this to determine the number of cells expressing GFP/CD8a (Total number of cells x % GFP^+ cells). Typically, approximately half of the GFP^+ cells are isolated by magnetic bead purification.

Day 14: $CD8a^+$ Cell Isolation

CD8a-expressing cells are isolated using the RoboSep™ Fully Automated Cell Separator (Catalog # 21000, 20119, 20155) and the Mouse CD8a Isolation II kit (Stem Cell Technologies Catalog No.18953) as follows:

14. Resuspend cells at 10 x 10^6 cells per 100 µl of RoboSep buffer supplemented with IL-2 (1:2000 of IL-2, **Table 1**) and place in a 14 ml polystyrene round bottom sterile test tube. Be careful not to get bubbles in cell suspension or on the sides of the tube, since this will make the isolation less efficient.

15. Turn on the RoboSep and pick the 1st quadrant and select the 1st program labeled Biotin Isolation. When working with more than one type of cell/donor use one quadrant per cell type for a total of 4 different cell types/donors per isolation. When prompted enter in amount of buffer used to resuspend the cells. If there is a low frequency of infection (less than 10%) enter twice the amount of buffer, which will result in a two-fold concentration of antibody and cocktail during the isolation.

16. Load the RoboSep carousel based on the directions from the machine (it will give a diagram indicating what type of tubes are required and what each tube will have at the end of the procedure). The Positive fraction tube (located in the magnet) will be the CD8a$^+$ cells. The negative fraction tube will be a mixed population of both infected and uninfected cells but the frequency of infected cells will be less than it was before isolation. This mixed population can be used for controls, or for additional CD8a$^+$ cell isolations, if additional cells are required.

Days 14 to 28: Cell Quiescence

Vortex the positive fraction tube from the RoboSep to remove cells and beads from the side of the tube and then centrifuge and remove RoboSep buffer. Resuspend at 1 x 10^6 cells per ml, in primary cell media with the addition of the maintenance cytokine **(Table 1)** cytokines depending on cell type. The cells are cultured at normal levels of cytokines for 5 days without changing the media or adding any additional cytokines. During this period the cells slowly use up the cytokines and cell growth slows. Since the cells will continue to expand at the beginning of this step down protocol, it is possible to start the step down phase in the middle of the expansion phase. For example, if you want the cells to expand for 7 days, place in normal media at 1 x 10^6 cells per ml, then 2 days later add more cytokine media to bring them to 1 x 10^6 cells per ml, and culture for 5 more days without changing media or cytokines to allow them to step down.

For the cells to enter quiescence the amount of cytokines need to be reduced to slow growth, mimicking what happens *in vivo* this is done by using the dilutions of cytokines listed in **Table 3**. Allow the cells to remain in this media for at least 1 week to allow the cells to fully enter

Table 3. Cytokines for Quiescence Medium

Subtype	Cytokine	Stock Conc.	Quiescence Media	Maintanence Media	Source	Catalog No.
Th1,<br>Th2,<br>TReg,	rIL-2<br>TGF-β1<br>IL-8<br>IL-10	15,000 IU/ml<br>10 µg/ml<br>50 µg/ml<br>10 µg/ml	1:4000<br>1:1000<br>1:1000<br>1:1000	1:4000	NIH<br>Preprotech<br>Preprotech<br>Preprotech	136<br>100-21C<br>200-08M<br>200-10
Th17	rIL-2<br>IL-23<br>TGF-β<br>IL-8<br>IL-10	15,000 IU/ml<br>12.5 µg/ml<br>10 µg/ml<br>50 µg/ml<br>10 µg/ml	1:4000<br>1:4000<br>1:1000<br>1:1000<br>1:1000	1:4000<br>1:4000	NIH<br>Preprotech<br>Preprotech<br>Preprotech<br>Preprotech	136<br>200-23<br>100-21C<br>200-08M<br>200-10

quiescence. The viability of your cells drops to about 60% due to apoptosis during entry into quiescence. Entry into quiescence should be monitored by cell cycle monitoring, typically by flow cytometry for EdU incorporation and CycB1 and CycD3 levels.

Optional Thy1.2 infection and Selection

Thy1.2 can be used as a selection marker using our pHR'-Nef+-Thy1.2-T2A-GFP virus. This vector uncouples GFP from the surface protein so that GFP can be used a HIV activation marker. Thy1.2 is also easier to purify resulting in a higher yield of HIV infected cells during isolation. To use this option, infect using the pHR'-Nef+-Thy1.2-T2A-GFP virus in the same manner as described for the pHR'-Nef+-CD8a/GFP virus. To select for Thy1.2 you can use the previous described protocol except use the EasySep™ Mouse CD90.2 Positive Selection Kit II (Stemcell, 18951RF).

EDITS Method

CD4 memory cells were negatively isolated using robotic magnetic bead isolation technology (Stemcell, 19157RF). 1.25 x 10^6 cells were stimulated with Dyna T cell simulation beads (1:1 ratio), 500 nM SAHA, 10 ng/ml IL-15, combination of IL-15 and SAHA or 10 ng/ml TNF-α for 24 hrs. Total RNA was isolated using Qiagen RNeasy purification system (Qiagen, 74134) following manufactures protocol. The entire sample was used as template in a one-step RT-PCR reaction (Thermoscientific, AB-4104A). Primers were designed to bind to either side of the HIV Env RNA splice junction using highly conserved regions of HIV and yielding a product of 168 bp. In addition to the priming sequence, the reverse primer has a synthetic GEX R-AATGATACGGCGACCACC sequence placed directly after the priming region to allow for further amplification using nested PCR. After cDNA synthesis and PCR, 2 µl of the reaction was used as template for a subsequent round of nested PCR using a high fidelity Phusion Flash polymerase (Thermoscientific, F548). To allow for NGS sequencing, Ion torrent A forward and Trp reverse adapters were added to the nested primer sets, as well as a unique barcode in the forward primer, to allow for multiplexing of samples. Samples were then pooled and primers were removed using GeneJET NGS cleanup kit (Fisher Scientific, FERK0852). DNA concentrations were measured

by a Qubit fluorescent reader and 300 pg of the pooled sample was then sequenced using an Ion Torrent Sequencing system following manufacture's protocol. Barcodes were separated by sample using the Ion Torrent Browser and all reads were filtered to remove short products (under 80 bp) and only reads that contained the GEX reverse sequence were retained. The filtered reads were then mapped to a synthetically spliced HXB2 sequence and total mapped reads were scored.

RNA-Seq Analysis

The RNA-Seq data sets, comprising two replicate studies performed over a year apart, have been submitted to the SRA database (accession number: SRP145508). All RNA-Seq data sets passed the quality control step which was performed using FastQC (Babraham Bioinformatics) (**Table 4**).

Alignment of reads

On average, ~45 million single end 100 nucleotide long reads were obtained for each sample. The reads were pre-processed for removal of adaptor-based sequences and low-quality nucleotides at the ends of the reads using Trim Galore (Babraham Bioinformatics), with reads maintaining at least a 30-nucleotide length after this step used for the alignment step. Alignment was performed using HISAT2 and hg38/GRCh38 version of the human genome, followed by calculation of the number of reads mapping to each gene using HTSeq-count and Gencode V27 transcriptome as the reference annotation. Gene expression values were calculated as TPM (transcripts per million cellular RNAs) and protein-coding genes with very low expression values (< 1 TPM) were excluded from the study.

To identify the reads mapping to the HIV genome, RNA-Seq reads were mapped to the the transcribed region of the HIV construct used in the present study in the context of the entire human genome (hg38) to minimize false alignment rate. Alignments using the entire sequence of the HIV constructs instead of the transcribed sequences yielded highly similar results. Normalization of read libraries and all pairwise differential expression tests were performed using the statistical package edgeR with polarization identity as the blocking variable when appropriate

Table 4. The RNA-seq datasets used in this study. The quality control results for each sample as calculated by FastQC is listed along with the biological conditions under which the sample was collected. The two technical replicate datasets, which were performed over a year apart, are indicated. Additional technical replicates were obtained for Treg cells under quiescent state in the first replicate dataset.

Library name	Polarization identity	HIV	Sample Collection	Group	Seq depth	Read length	GC %
Th17A	Th17	Yes	24 h after reactivation	2	63011881	101	47
Th17E	Th17	Yes	72 h after infection	2	48665302	101	49
Th17EM	Th17	Mock	72 h after infection	2	57513014	101	50
Th17Q	Th17	Yes	Quiescent	2	40899143	101	49
Th1A	Th1	Yes	24 h after reactivation	2	40674868	101	48
Th1E	Th1	Yes	72 h after infection	2	47371467	101	49
Th1EM	Th1	Mock	72 h after infection	2	58323705	101	48
Th1Q	Th1	Yes	Quiescent	2	59320263	101	49
Th2A	Th2	Yes	24 h after reactivation	2	48845130	101	46
Th2E	Th2	Yes	72 h after infection	2	46308516	101	50
Th2EM	Th2	Mock	72 h after infection	2	49243198	101	49
Th2Q	Th2	Yes	Quiescent	2	49244968	101	50
TregA	Treg	Yes	24 h after reactivation	2	55133380	101	49
TregE	Treg	Yes	72 h after infection	2	47769377	101	49
TregEM	Treg	Mock	72 h after infection	2	45565098	101	50
TregQ	Treg	Yes	Quiescent	2	43566417	101	49
Th17A	Th17	Yes	24 h after reactivation	1	33829282	101	49
Th17E	Th17	Yes	72 h after infection	1	40764059	101	48
Th17Q	Th17	Yes	Quiescent	1	32607576	101	48
TregA	Treg	Yes	24 h after reactivation	1	37986599	101	48
TregE	Treg	Yes	72 h after infection	1	39659763	101	48
TregQ	Treg	Yes	Quiescent	1	35026777	101	48
TregQ	Treg	Yes	Quiescent	1	35073317	101	48
TregQ	Treg	Yes	Quiescent	1	36000000	101	48
TregQ	Treg	Yes	Quiescent	1	36000000	101	48

and false discovery rate (FDR) values were calculated for each differential expression value. Genes were considered differentially expressed when the calculated expression level change was >=2 fold with FDR<0.05.

Normalized read histograms (**Figs. 3C and 3D**) were generated using the R package GVIZ (Biocoductor).

Pathway analysis

The positive- and negatively enriched pathways and genesets were identified using the HALLMARK module of the MSigDB V6 database of gene sets. Enrichment levels, p-values and FDR were calculated using the statistical tool GSEA using the weighted scoring scheme and the

number of permutations set to 1000 with the input gene lists ranked based on their differential expression value while controlling the false discovery rate.

Principal component analysis

Principal component analysis, which provides an unbiased method for clustering of RNA-Seq samples based on their overall pattern of gene expression, was performed using the decomposition module of the Scikit-learn library of Python.

Heat maps on RNA-seq data

Normalized gene-level expression values were used to draw the heat maps with row scaling when appropriate, while controlling for high gene-specific dispersion within replicate groups.

qPCR RNA Array

At each step of the QUECEL process, 1 x 10^6 cells were collected and RNA was isolated using RNeasy Mini kits (Qiagen, 74104) following the manufactures protocol. cDNA was produced using the RT2 HT First Strand Kit (Qiagen, 330411) with 0.5 µg of RNA used a template. The cDNA reaction was added directly to the RT² Profiler™ PCR Array Human T Helper Cell Differentiation array (Qiagen, 330231) or the RT² Profiler™ PCR Array Human Th17 Response array (Qiagen, 330231) using LightCycler® FastStart DNA Master SYBR Green I (Roche, 03003230001) as master mix following manufactures protocol. Cycling was performed on a BioRad MyIQ2 using the following cycling protocol: 1 cycle for 10 minutes at 95° C, 40 cycles of 15 sec at 95° C, 30 sec at 55° C and 30 sec at 72° C with fluorescence data collection.

Ct values were calculated and imported into the SA Biosciences PCR Array Data Analysis Excel Template for each assay type, which created scatter plots and raw values for heat map generation.
